# Reliability and reproducibility of systematic reviews informing the 2020–2025 Dietary Guidelines for Americans: a pilot study

**DOI:** 10.1016/j.ajcnut.2024.10.013

**Published:** 2024-12-12

**Authors:** Alexandra M Bodnaruc, Hassan Khan, Nicole Shaver, Alexandria Bennett, Yiu Lin Wong, Catherine Gracey, Valentina Ly, Beverley Shea, Julian Little, Melissa Brouwers, Dennis Bier, David Moher

**Affiliations:** 1 School of Epidemiology and Public Health, Faculty of Medicine, University of Ottawa, Ottawa, ON, Canada; 2 Clinical Epidemiology Program, Ottawa Hospital Research Institute, Ottawa, ON, Canada; 3 School of Chinese Medicine, Hong Kong Baptist University, Hong Kong SAR, China; 4 Dalhousie University, Halifax, NS, Canada; 5 University of Ottawa, Ottawa, ON, Canada; 6 Children’s Nutrition Research Center, Baylor College of Medicine, Houston, TX, United States

## Abstract

**Background:**

Although high-quality nutrition systematic reviews (SRs) are important for clinical decision making, there remains debate on their methodological quality and reporting transparency.

**Objectives:**

The objective of this study was to assess the reliability and reproducibility of a sample of SRs produced by the Nutrition Evidence Systematic Review (NESR) team to inform the 2020–2025 Dietary Guidelines for Americans (DGAs).

**Methods:**

We evaluated a sample of 8 SRs from the DGA dietary patterns subcommittee for methodological quality using the Assessment of Multiple Systematic Reviews 2 (AMSTAR 2) tool and for reporting transparency using the PRISMA 2020 and PRISMA literature search extension (PRISMA-S) checklists. We assessed the quality and reproducibility of the original search strategy of one selected SR using the Peer Review of Electronic Search Strategies checklist. The reporting transparency of the SR’s narrative data synthesis was assessed using the Synthesis Without Meta-Analysis (SWiM) checklist. Interpretation bias was evaluated using existing spin bias classifications in systematic reviews.

**Results:**

The AMSTAR 2 assessment identified critical methodological weaknesses, and all included SRs were judged to be of critically low quality. Overall, 74% of the PRISMA 2020 checklist items and 63% of the PRISMA-S checklist items were satisfactorily fulfilled. We identified several errors and inconsistencies in the search strategy and could not reproduce searches within a 10% margin of the original results. The SWiM assessment identified concerns regarding the reporting transparency of the narrative data synthesis, but the spin bias assessment revealed no evidence of interpretation bias.

**Conclusions:**

Several methodological quality and reporting concerns were identified, which could lead to reliability and reproducibility issues should a full reproduction attempt be made. However, additional research is needed to confirm the impact of these findings on conclusions statements and their generalizability across the NESR team SRs.

This study was registered in the Open Science Framework (https://osf.io/ns6a9/).

## Introduction

Optimally, clinical practice guidelines (CPGs) are systematically developed, evidence-based recommendations designed to assist various players, including national organizations, health care providers, policymakers, patients, and others, in making well-informed health care and public health policy decisions [1]. It is widely recognized that high-quality systematic reviews (SRs) play a crucial role in consolidating the latest and most reliable evidence, enabling the development of reliable and trustworthy recommendations [1,2]. Given the widespread production and utilization of CPGs, it is imperative that the SRs informing them adhere to rigorous methodological standards [3]. Several reporting guidelines have been established to ensure transparency and reproducibility, enabling others to reproduce or update previous SRs and aiding decision makers in evaluating the credibility of the SRs’ findings [[4], [5], [6], [7], [8], [9], [10], [11], [12], [13]]. However, despite the availability of such guidance, SR authors often fall short in implementing these reporting recommendations, resulting in suboptimal research practices for reproducibility, particularly within the field of nutrition [[14], [15], [16], [17]].

Established in the 1980s by the United States Department of Health and Human Services and the USDA, the Dietary Guidelines for Americans (DGAs) are dietary recommendations purportedly aimed at helping Americans meet nutritional needs, promote health, and prevent chronic diseases across the lifespan [[18], [19], [20]]. The recommendations of the DGA serve as the cornerstone for federal nutrition programs and policies within the United States and are mandated by law to be updated at least every 5 y to best reflect prevailing scientific evidence. Given their implications, the robustness of the scientific process underpinning the DGA is of paramount importance. Over the past 40 y, several researchers have expressed concerns about the adequacy of the scientific evidence informing the DGA [[21], [22], [23], [24], [25]]. These concerns were corroborated by a report mandated by the United States Congress and conducted by the National Academies of Sciences, Engineering, and Medicine [26]. The report highlighted specific concerns regarding scientific methodology, scientific rigor, and transparency [27,28].

The development process of the DGA has evolved over time to better align with advances in nutrition sciences, public health, and best practices in scientific review and guidelines development. The development process for the 2020–2025 DGA included 4 stages, namely *1*) identifying the topics to be examined and the supporting scientific questions; *2*) appointing a DGA Committee (DGAC) to review current scientific evidence and develop a scientific report; *3*) developing the new edition of the DGA; and *4*) implementing the DGA through federal and nonfederal programs [18]. The DGAC answers each scientific question using analyses of national data sets, modeling of food patterns, and SRs produced by the Nutrition Evidence Systematic Review (NESR) team [29,30]. The NESR team includes a diverse group of SR and methodological experts (e.g., SR analysts and information specialists) alongside content experts with advanced degrees in nutrition, public health, and epidemiology. Public and patient involvement is also evident throughout the SR process, from identifying high-priority SR questions and developing the SR protocol to grading the strength of evidence and formulating conclusion statements. For the most recent iteration of the DGA, the DGAC included 6 subcommittees focusing on different topics, namely dietary patterns, pregnancy and lactation, birth to age 24 mo, beverages and added sugars, dietary fats and seafood, and frequency of eating [31]. The NESR team conducted 33 SRs to assist the DGAC in developing the 2020–2025 DGA.

In light of the concerns mentioned above, this pilot study aimed to evaluate the reliability and reproducibility of a sample of SRs conducted by the NESR team to inform the DGA. More specifically, our research team aimed to answer the following key questions (KQs):

1. Do the SRs conducted by the NESR team report a transparent, complete, and accurate account of their SR process such that they are reliable and reproducible?
2. If a SR was reproduced, would any observed differences from replicating the search strategy and data analysis change the original conclusions made by the NESR team?

## Methods

This pilot study was conducted between March 2023 and April 2024 by the Knowledge Synthesis and Application Unit at the School of Epidemiology and Public Health, University of Ottawa. A protocol was developed and registered in the Open Science Framework (OSF) (https://osf.io/ns6a9/). In keeping with open science practices, all study materials are provided in the OSF project folder. This study is reported following the PRISMA 2020 statement (Supplementary Files 1 and 2) [5].

### Protocol amendments

Minor amendments were made to the protocol. The PRISMA-S checklist was initially intended to be applied only for KQ2. The PRISMA-S checklist was also used to assess all SRs included in KQ1 to strengthen our conclusions.

### Assessment of the methodological quality and reporting transparency of a sample of the NESR team’s SRs (KQ1)

To evaluate methodological quality and reporting transparency, we selected the DGA subcommittee for which the NESR team conducted the highest number of SRs. The dietary patterns subcommittee used 8 SRs, the pregnancy and lactation subcommittee used 7 SRs, the birth to 24 mo subcommittee used 4 SRs, the beverages and added sugars subcommittee used 7 SRs, the dietary fats and seafood subcommittee used 3 SRs, and the frequency of eating subcommittee used 5 SRs. Therefore, for KQ1, we evaluated the sample of 8 SRs from the DGA dietary patterns subcommittee [[32], [33], [34], [35], [36], [37], [38], [39]].

#### Assessment of the methodological quality

To evaluate methodological quality, 2 independent reviewers (HK and YLW) applied the Assessment of Multiple Systematic Reviews 2 (AMSTAR 2) tool to the 8 SRs [4]. Conflicts were discussed and resolved by consensus or consulting with a senior investigator (DM). AMSTAR 2 is a critical appraisal tool to assess the overall methodological quality of SRs [4]. It was used to systematically interpret reliability and overall confidence in the results of each SR as well as to identify any critical areas of weakness. The AMSTAR 2 tool has been found to be valid and has moderate interrater reliability [40,41]. Prior to the quality assessment, 2 independent reviewers completed a pilot exercise on a randomly selected SR from the dietary patterns DGA subcommittee. The AMSTAR 2 assessment was summarized visually, and any identified weaknesses among its critical domains are described in the results section.

#### Assessment of the reporting transparency

To evaluate reporting transparency, 1 reviewer (HK) applied the PRISMA 2020 checklist to the 8 SRs [5]. A second independent reviewer (YLW) verified the assessment. Conflicts were discussed and resolved by consensus or consulting with a senior investigator (DM). The PRISMA 2020 checklist is a comprehensive SR and meta-analysis reporting guideline, providing authors with the necessary guidance to prepare a transparent, comprehensive, and precise explanation of the purpose behind the SR, the methodologies employed, and the findings obtained [5]. The PRISMA 2020 assessment was summarized visually, and unfulfilled items are discussed in the results section.

#### Assessment of the reporting transparency of the search strategy

To evaluate the reporting transparency of the search strategy, 1 reviewer (HK) applied the PRISMA-S checklist to all 8 SRs [7]. A second independent reviewer (AMB) verified the assessment. Conflicts were discussed and resolved by consensus or consulting with a senior investigator (DM). The PRISMA-S checklist is an extension of the PRISMA 2020 statement, providing authors with the necessary guidance to prepare and report a transparent and comprehensive literature search strategy [7]. The PRISMA-S assessment was summarized visually, and unfulfilled items are discussed in the results section.

#### Data analysis

For KQ1 analysis, we aimed to evaluate if the NESR team reported a transparent, complete, and accurate account of their SR process. To inform this judgment, we summarized and critically evaluated our results from the AMSTAR 2, PRISMA 2020, and PRISMA-S assessments. The sampled SRs were judged to have low reliability and reproducibility if the tools identified critical flaws in the methodological quality or a lack of transparency in the reporting of the SRs.

### Assessment of the reproducibility of one SR conducted by the NESR team (KQ2)

To evaluate reproducibility, we selected one SR from the dietary patterns DGA subcommittee, namely the SR on dietary patterns and neurocognitive health [37]. This SR was selected a priori because it was assessed in KQ1 and included a reasonable number of studies (e.g., *n* = 26) to consider for reproduction. Other SRs in this category included too few (e.g., *n* = 4–9) or too many (e.g., *n* = 66–190) studies.

#### Assessment of the search strategy

We worked with a senior information specialist (VL) to peer review the NESR team’s original search strategy using the Peer Review of Electronic Search Strategies (PRESS) checklist [42]. To assess the completeness of the search strategy as part of the PRESS review, we asked the information specialist to identify any additional databases or grey literature sources that may have been missed in the original search. The reporting transparency of the original search strategy was also reviewed using the PRISMA-S checklist [7]. With the help of the information specialist, we also determined if the search strategy was reproducible by rerunning the strategy as described in the NESR team’s SR. Based on methods from recent meta-research on the topic of reproducibility of SRs [43], the search strategy was considered reproducible if all the database searches could be reproduced within 10% of the original SR numbers.

#### Assessment of the data synthesis and analysis methods

All of the NESR team’s SR syntheses were narrative. They were performed using a predefined process involving the NESR team summarizing the evidence and the DGA subcommittee members writing the conclusion statements. Therefore, we assessed the reporting transparency of their narrative analysis using the SWiM checklist [9]. The SWiM checklist uses 9 reporting items to evaluate the reporting transparency of SRs that synthesize data narratively without meta-analysis [9]. One reviewer (HK) applied the SWiM checklist to the SR, and a second independent reviewer (AMB) verified the assessment. Conflicts were discussed and resolved by consensus or consulting with a senior investigator (DM).

We evaluated if there were sufficient data to undertake meta-analyses for observational studies. To identify if ≥3 studies were similar enough to have their results pooled, we created an analytic framework using the ‘population, intervention, comparator, outcome, timing’ (PICOT) approach. Studies were separated by type of outcome measure (i.e., dichotomous or continuous outcomes) and then by type of dietary exposure (i.e., dietary guideline-related indices/scores, Mediterranean diet-related indices/scores, Dietary Approaches to Stop Hypertension diet indices/scores, or other indices/scores). This process facilitated the comparison of each element of the PICOT framework to explore potential drivers of clinical heterogeneity across studies. Studies were deemed eligible for meta-analysis when they were similar enough across the PICOT framework elements. For these studies, random-effects pooling was performed using R Studio Software, version 2023.12.1 [44] with the “meta” package [45]. Forest plots were generated to visualize the results. The between-study heterogeneity was calculated using the *I*^2^ statistic [46,47]. The *I*^2^ test value corresponds to the percentage ratio of between-study variance over the sum of between- and within-study variances [46,47]. Values ranging between 0 and 40%, 40% and 60%, 60% and 90%, and values >90% were interpreted as low, moderate, substantial, and considerable heterogeneity, respectively [46,47]. To obtain an objective third-party opinion on whether a meta-analysis could have been executed on the original NESR team’s results, we also consulted with a methodological expert on meta-analyses (BH).

Any quantitative meta-analysis results were compared with the conclusions drawn by the original SR authors in their narrative syntheses. We compared any observed differences in the direction and magnitude of results between the original SR and the reproduced steps of the SR.

#### Assessment of interpretation bias

We evaluated the interpretation of results from the NESR team’s SR by assessing spin bias. Spin bias is the intentional or unintentional interpretation of research results that fails to reflect the nature and range of findings and can result in misleading conclusions. We assessed spin bias using the methods outlined by Yavchitz et al. (2016) [48], which include misleading reporting, misleading interpretation, and inappropriate extrapolation. One reviewer (HK) applied the Yavchitz et al. [48] tool to the NESR team’s SR. A second independent reviewer (AMB) verified the assessment. Conflicts were resolved by consensus or by consulting with a senior investigator (DM).

#### Data analysis

For KQ2, we aimed to answer if any observed differences from reproducing the search strategy and data analysis would change the original conclusions made by the NESR team. To evaluate the reporting transparency, quality, and reproducibility of the search strategy, we examined the results of the PRISMA-S and PRESS checklists. For the reproduced search results, we also considered a change in the number of records >10% between the original and replicated results to be a substantive difference. To assess the reproducibility of the analysis, we first examined the results of the SWiM assessment and spin bias evaluation to determine the quality of the interpretation and the reporting of the results in the selected SR. We subsequently performed a meta-analysis and compared synthesized results between the NESR team’s SR and our reproduced findings.

## Results

To answer KQ1 and KQ2, information was gathered from both the published SRs [[32], [33], [34], [35], [36], [37], [38], [39]] and the NESR team’s methodology manual [49]. Some information, such as methods used for extracting data, assessing the risk of bias of each included study, synthesizing the evidence, developing conclusion statements, and grading the evidence underlying the conclusion statements were only described in the NESR team’s methodology manual.

### KQ1 results

#### Assessment of the methodological quality using AMSTAR 2

**FIGURE 1:**
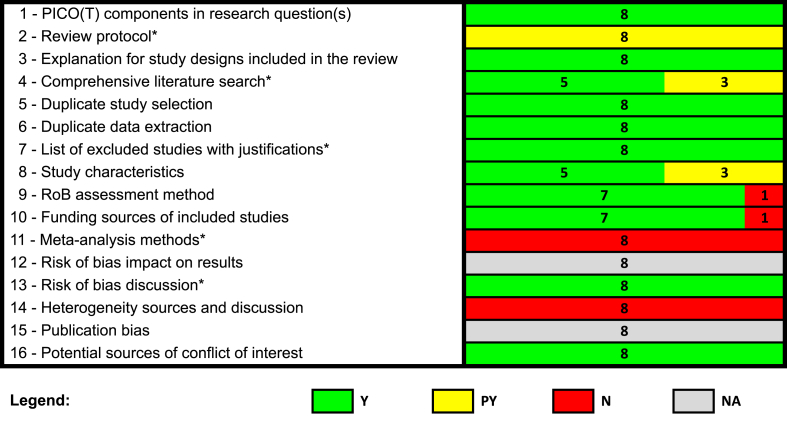
Summary of AMSTAR 2 assessments of the NESR team SRs on dietary patterns and health outcomes. (∗) indicates a critical domain. Abbreviations: AMSTAR 2, Assessment of Multiple Systematic Reviews 2; N, no; NA, not applicable; NESR, Nutrition Evidence Systematic Review; PICO(T), Population, Intervention, Comparator, Outcome, Timing; PY, possible yes; RoB, risk of bias; SR, systematic review; Y, Yes.

The methodological quality of all 8 evaluated SRs in the nutrition patterns DGA subcommittee was rated as critically low (Figure 1; Supplemental File 3). Despite including 2 SR analysts in their team (and in the list of authors), the quality of all evaluated SRs was downgraded for the absence of meta-analyses and no justification for this absence. The NESR team determined a priori that all data synthesis would be narrative. Therefore, contrary to the approach recommended by Cochrane Methods, the appropriateness of conducting meta-analyses was not evaluated based on the SR results [46]. As shown in Figure 1, several other items were rated as unmet (i.e., “no” ratings) or partially unmet (i.e., “partial yes” ratings). For example, all SRs only partially met item #2 because their protocol was not registered and did not contain a plan for conducting meta-analyses or investigating heterogeneity.

#### Assessment of the reporting transparency using the PRISMA 2020 checklist

**FIGURE 2:**
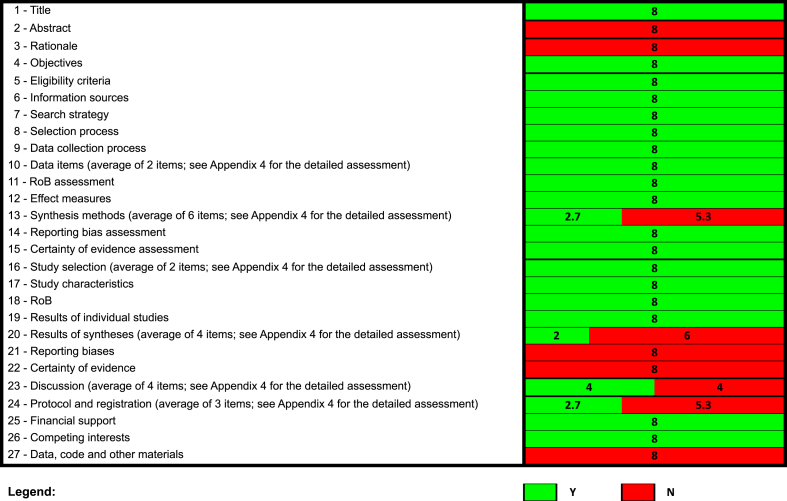
Summary of PRISMA 2020 assessments of the NESR team SRs on dietary patterns and health outcomes. N, no; NESR, Nutrition Evidence Systematic Review; RoB: risk of bias; SR, systematic review; Y, yes.

None of the 8 evaluated SRs mentioned using the PRISMA checklist for their reporting. The reporting in general was very similar across all assessed SRs; there was no variation in item reporting patterns between SRs (Figure 2; Supplemental File 4). Across all 8 SRs, 20 of the 27 items (74%) on the PRISMA 2020 checklist were satisfactorily fulfilled, whereas 7 items (26%) were either unfulfilled or only partially fulfilled. Unfulfilled items were the abstract (item #2), the rationale (item #3), and the availability of data, code, and other materials (item #27). Partially fulfilled items were the synthesis methods (item #13), the results of syntheses (item #20), the discussion (item #23), and the registration and protocol (item #24).

With regard to item #13, all SRs lacked a description of any methods required to prepare the data for presentation or synthesis, any methods used to synthesize results, any methods used to explore possible causes of heterogeneity among study results, and any sensitivity analyses conducted to assess the robustness of the synthesis results. As a consequence of no meta-analyses being conducted, the SRs did not present the results of any statistical syntheses, investigations of possible causes of heterogeneity, and sensitivity analyses. For the discussion, although limitations in the quality and overall certainty of evidence were discussed, all SRs did not provide a general interpretation of the results in the context of other evidence and did not discuss the limitations of the actual methodological processes used. For item #24, all SRs did not provide registration information or explain any amendments to information provided at registration or in the protocol.

#### Assessment of the reporting transparency of the search strategy using the PRISMA-S checklist

**FIGURE 3:**
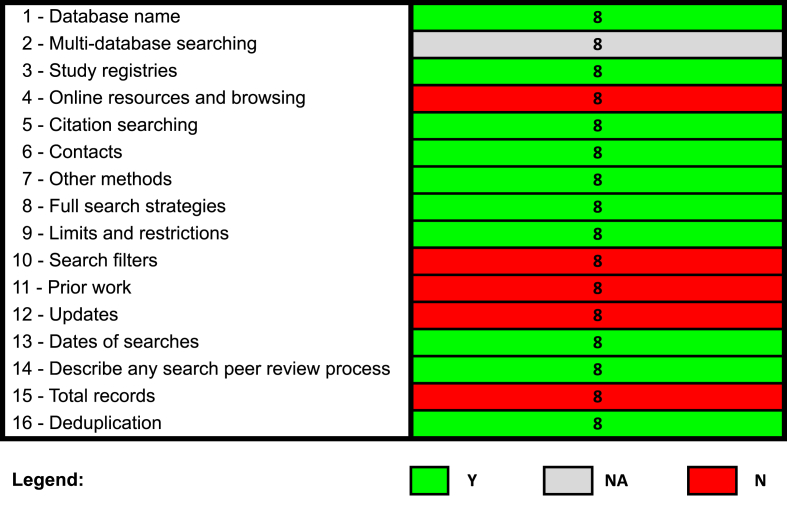
Summary of PRISMA-S checklists for the NESR team SRs on dietary patterns and health outcomes. N, no; NA, not applicable; NESR, Nutrition Evidence Systematic Review; SR, systematic review; Y, yes.

All 8 SRs included 2 information specialists as part of the SR team (and the list of authors). Across all 8 SRs, 10 of the 16 PRISMA-S checklist items (63%) were satisfactorily fulfilled, 5 items (31%) were unfulfilled, and 1 item (6%) was not applicable (Figure 3; Supplemental File 5). All SRs did not report if any online or print source was searched or browsed (item #4), if search filters were used (item #10), if any search strategies from prior SRs were adapted or reused (item #11), and any methods used to update the searches (item #12). SRs also did not document the total number of records identified from each database or other information sources (item #15). Although some of these items may not have been applicable to the evaluated SRs, we did not have enough information to judge if they were not applicable or not reported.

#### Data analysis

For KQ1, we aimed to evaluate reliability and reproducibility by assessing whether the NESR team reported a transparent, complete, and accurate account of their SR process. To that end, the methodological quality and reporting transparency of a sample of 8 SRs in the dietary patterns DGA subcommittee was evaluated.

Assessments were found to be highly homogeneous across SRs, suggesting that similar processes were used for planning, conducting, and reporting the SRs. The AMSTAR 2 assessments indicated critical weaknesses in the conduct of the SRs, most notably related to the justification of synthesis and analysis methods. The overall reporting of the SRs and the reporting of the SR literature search strategy, as assessed with PRISMA 2020 and PRISMA-S checklists, respectively, were found to be suboptimal, contributing to lowering the SRs’ reliability and potentially leading to reproducibility issues.

### KQ2 results

#### Assessment of the search strategy

The search strategies for PubMed (pubmed.ncbi.nlm.nih.gov), Embase (embase.com), and Cochrane Central Register of Controlled Trials (CENTRAL, Wiley) were analyzed as described in the selected SR by CG on 3 and 4 August, 2023 and verified by VL. Searches were analyzed from 2014 to 2020 to align with the original search dates. However, we were unable to limit searches to the exact date in 2020 that the NESR ran their searches, which may have led to some discrepancy in the number of records obtained.

After rerunning the search results, our team identified a large discrepancy in the number of records retrieved compared with those reported in the original SR (Supplemental File 6). The reproduced searches yielded 10,201 results (not deduplicated) across the 3 electronic databases (Embase, *n* = 6768; PubMed, *n* = 2481; CENTRAL, *n* = 952) (see PRISMA flowchart in Supplemental File 6). The initial NESR team’s SR search results yielded 5621 records, with 3550 records left after the removal of 2071 duplicate records. As our team described in the PRISMA-S assessment, the authors did not document the total number of records identified from each database and other information sources (PRISMA-S item #15), which made it difficult to determine which database(s) varied in the number of records retrieved. Although changes in the platform used to search electronic databases and the date may have partially influenced the number of records retrieved, this is unlikely to fully explain the discrepancy in the number of records.

The PubMed search strategy was peer-reviewed by CG and VL. Based on the PRESS assessment, it was found that there were errors and inconsistencies in the search strategy that would affect the retrieved results (Supplemental File 7). The most notable issues were the lack of quotation marks when searching multiword terms, as well as missing field codes for title and abstract, which would result in PubMed’s automatic term mapping. The use of filters to exclude specific publication types (e.g., editorials, comments, retracted publication, retraction notices, and systematic and narrative reviews) at the database searching stage of the SR is also not recommended because it can lead to excluding potentially relevant records.

#### Assessment of the data synthesis and analysis methods

Of the 9 SWiM items, only 1 (i.e., item #8 - Reporting results) was entirely reported by the NESR team. As shown in Table 1, all remaining items were not explicitly reported in the manuscript. When an item was not reported in the SR methodology, we consulted the DGA methodology manual to search for additional supporting information [30]. Some additional descriptions of typical methods used by the NESR team were provided in the methodology manual for items #1, #3, #4 and #7. Although this additional information provided some insight into the typical NESR team’s methods, there remained a lack of description and justification on how these methods were applied within this specific SR. For example, for item #1, the synthesis appears to have been grouped based on study design, intervention assessment (e.g., indices/scores), and statistical significance, but no description or rationale was provided to support this decision. Importantly, the authors did not justify their decision to conduct a narrative synthesis (item #3) or acknowledge the limitations of a narrative synthesis approach (item #9).

**TABLE 1:** Synthesis without meta-analysis reporting items for the NESR team SR on dietary patterns and neurocognitive health.

SWiM item	Item description	Page where item is reported	Other comments
Methods			
1. Grouping studies for synthesis	1a) Provide a description of, and rationale for, the groups used in the synthesis (e.g., groupings of populations, interventions, outcomes, study design).	NR	Although we observe that studies are grouped by study design, no description and rationale is provided. Although no clear description or rationale is provided, information on this item is also reported on pages 15 (“create evidence tables”) and 19 (“synthesize the evidence”) of the *Scientific Report of the 2020 DGAC: Advisory Report to the Secretary of Agriculture and the Secretary of Health and Human Services*.
	1b) Detail and provide rationale for any changes made subsequent to the protocol in the groups used in the synthesis.	NR	NA
2. Describe the standardized metric and transformation methods used	Describe the standardized metric for each outcome. Explain why the metric(s) was chosen, and describe any methods used to transform the intervention effects, as reported in the study, to the standardized metric, citing any methodological guidance consulted.	NR	NA
3. Describe the synthesis methods	Describe and justify the methods used to synthesize the effects for each outcome when it was not possible to undertake a meta-analysis of effect estimates.	NR	The main paper reports that the results will be narratively synthesiszed (page 11), but no rationale is provided. Although no clear justification is provided, some additional information is reported on pages 15 (“create evidence tables”) and 19 (“synthesize the evidence”) of the *Scientific Report of the 2020 DGAC: Advisory Report to the Secretary of Agriculture and the Secretary of Health and Human Services*.
4. Criteria used to prioritize results for summary and synthesis	Where applicable, provide the criteria used, with supporting justification, to select the particular studies, or a particular study, for the main synthesis or to draw conclusions from the synthesis (e.g., based on study design, risk of bias assessments, directness in relation to the SR question).	NR	The main paper reports the inclusion and exclusion criteria (pages 51–52), but no rationale is provided.
5. Investigation of heterogeneity in reported effects	State the method(s) used to examine heterogeneity in reported effects when it was not possible to undertake a meta-analysis of effect estimates and its extensions to investigate heterogeneity.	NR	The main paper reports that “consistency” of results was assessed, but no description of the methods is provided.
6. Certainty of evidence	Describe the methods used to assess certainty of the synthesis findings.	NR	The main paper reports that the strength of evidence was assessed using pre-established criteria for risk of bias, consistency, directness, precision, and generalizability (page 11), but no description of the methods is provided. Although no description of the methods is provided, information on this item is also reported on pages 20–23 of the of the *Scientific Report of the 2020 DGAC: Advisory Report to the Secretary of Agriculture and the Secretary of Health and Human Services*.
7. Data presentation methods	Describe the graphical and tabular methods used to present the effects (e.g., tables, forest plots, harvest plots). Specify key study characteristics (e.g., study design, risk of bias) used to order the studies in the text and any tables or graphs, clearly referencing the studies included.	NR	The main paper does not explicitly report data presentation methods. Nonetheless, we observe that the authors used tables to present the effects, separated studies by design, and presented studies in alphabetical order (pages 26–46). Although data presentation methods are not explicitly reported, information on this item is also reported on page 15 (“create evidence tables”) and 19 (“synthesize the evidence”) of the *Scientific Report of the 2020 DGAC: Advisory Report to the Secretary of Agriculture and the Secretary of Health and Human Services*.
Results			
8. Reporting results	For each comparison and outcome, provide a description of the synthesized findings and the certainty of the findings. Describe the result in language that is consistent with the question the synthesis addresses and indicate which studies contribute to the synthesis.	13–21	NA
Discussion			
9. Limitations of the synthesis	Report the limitations of the synthesis methods used and/or the groupings used in the synthesis and how these affect the conclusions that can be drawn in relation to the original SR question.	NR	The main paper describes the limitations of the body of evidence, but not the limitation of the SR methods per se.

Abbreviations: DGAC, Dietary Guidelines Advisory Committee; NA, not applicable; NESR, Nutrition Evidence Systematic Review; NR, not reported; SR, systematic review.

**FIGURE 4:**
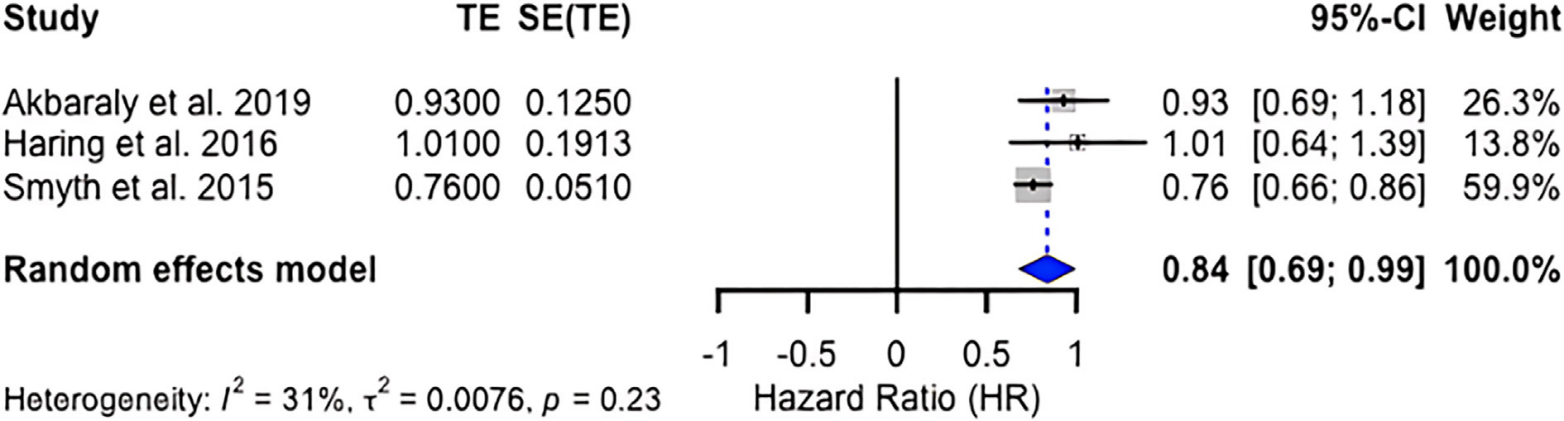
Random-effect model forest plot showing the association between AHEI score and neurocognitive decline. The sample sizes of the studies included in the meta-analysis were 8225 (Akbaraly et al., 2019 [50]), 6425 (Haring et al., 2016 [51]), and 27,860 (Smyth et al., 2015 [52]) participants. AHEI, Alternative Healthy Eating Index; CI, confidence interval; SE, standard error; TE, treatment effect.

**FIGURE 5:**
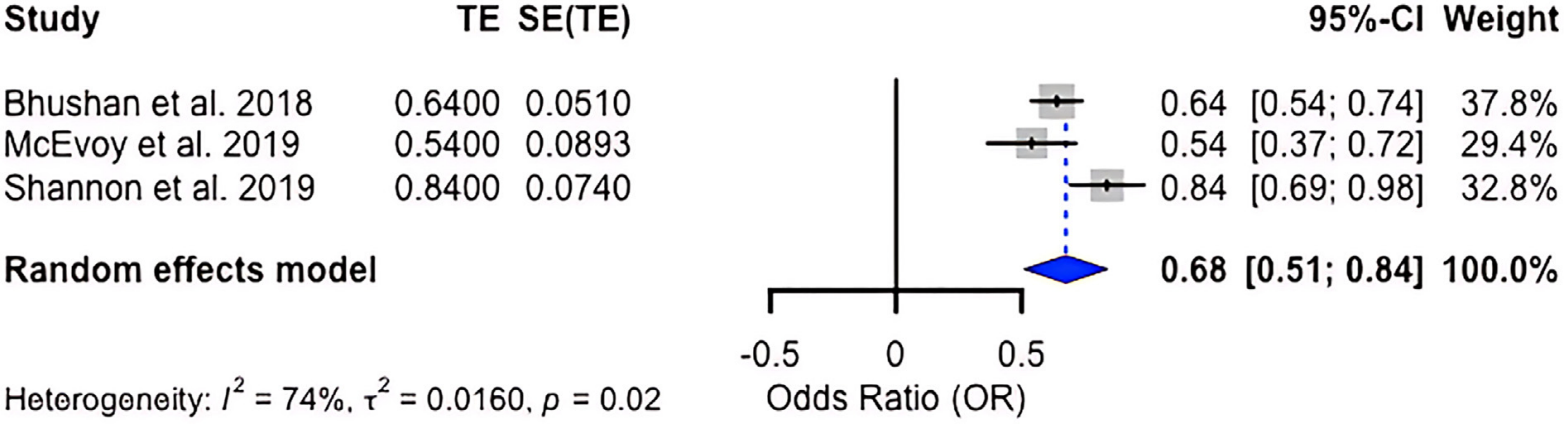
Random-effect model forest plot showing the association between Mediterranean diet and neurocognitive decline. The sample sizes of the studies included in this meta-analysis were 27,842 (Bhushan et al., 2018 [53]), 2621 (McEvoy et al., 2019 [54]), and 8009 (Shannon et al., 2019 [55]) participants. CI, confidence interval; SE, standard error; TE, treatment effect.

To visually illustrate the direction and magnitude of associations between dietary patterns and neurocognitive decline as well as to assess heterogeneity, we pooled effect estimates from 6 longitudinal cohort studies. For the association between the Alternative Healthy Eating Index (AHEI) and cognitive decline (3 studies [[50], [51], [52]]), the results indicated a hazard ratio of 0.84 (95% confidence interval [CI]: 0.69, 0.99) and an *I*^2^ statistic of 31% (*P* = 0.23), indicating moderate heterogeneity (see Figure 4). For the association between the Mediterranean diet and cognitive decline (3 studies [[53], [54], [55]]), results indicated an odds ratio of 0.68 (95% CI: 0.51, 0.84) and an *I*^2^ statistic of 74% (*P* = 0.02), indicating substantial heterogeneity (see Figure 5).

Based on the results of our meta-analyses and the opinion of the methodological expert on meta-analyses (BH) who was consulted by our research team, we concluded that, for most outcomes, a meta-analysis was likely not appropriate in the original SR due to *1*) the limited number of studies available for each exposure (i.e., dietary pattern) and *2*) the presence of clinical and statistical heterogeneity that could not be sufficiently explained via subgroup analyses or meta-regression. In summary, we confirmed the NESR team’s judgment of significant clinical heterogeneity among the included studies. Despite this heterogeneity, results were relatively consistent across studies, supporting inverse associations between healthy dietary patterns and adverse cognitive outcomes. Our quantitative findings align with the narrative description of the results provided by the NESR team. The original authors did not narratively describe the magnitude of associations but did report that the results were relatively consistent among studies with significant findings, suggesting that consuming healthier dietary patterns during adulthood was associated with improved cognitive outcomes or lower risk of cognitive impairment later in life. The authors also acknowledged the presence of heterogeneity among the results and the overall limited certainty of the available evidence due to the high risk of bias among the included studies.

#### Assessment of interpretation bias

Of the 28 items in the checklist presented by Yavchitz et al. [48], 20 were rated as “suggesting no evidence of spin bias” (Table 2). The remaining items were not applicable (6/28) or did not contain enough information to judge (2/28). For the abstract, of the 21 tool items, 14 were rated as suggesting no evidence of spin bias, 4 as not applicable, and 3 as not enough information to judge. We did not find evidence of misleading reporting, misleading interpretation, or inappropriate extrapolation in the abstract or main text. In the main text, the authors appeared to summarize all results narratively in a relatively consistent manner. Results were grouped by statistical significance within dietary patterns, and the overall magnitude of the effect of each primary study was not narratively interpreted. The actual results values from each study were included, and there was no disproportionate focus on *P* values alone. The authors did comment on the direction of results (benefits or harms) and consistency between estimates, but this was not judged to be inappropriate. In their overall summary statements, the NESR team reported limited certainty in the evidence and, therefore, in the overall conclusions drawn from the SR. The overall limitations in the evidence were acknowledged in the summary statements as well as in the text.

**TABLE 2:** Spin bias assessment (Yavchitz et al., 2016 [48]) for the NESR team SR on dietary patterns and neurocognitive health.

Category	Item description	Rating	Notes
Misleading reporting			
1	Failure to acknowledge a departure from protocol that could modify the interpretation of results	Not enough information to judge	The methodology section of the main manuscript (page 4) reports that there was a shared protocol and that public feedback was solicited. There is no link to the protocol or any mention of deviation from the protocol.
2	Selective reporting of or overemphasis on efficacy outcomes favoring the beneficial effect of the experimental intervention (e.g., secondary outcomes, subgroup analyses)	No evidence of spin bias	NA
3	Selective reporting of or overemphasis on harm outcomes favoring the safety of the experimental intervention	No evidence of spin bias	NA
4	No or inadequate reporting of the limitations of the SR	No evidence of spin bias	The main manuscript reports that lack of RCTs, differences in testing methods for the outcome, as well as validity and reliability as limitations of the SR.
5	Selective citation of articles in favor of the beneficial effect of the experimental intervention	No evidence of spin bias	NA
6	Authors hide or do not present any conflict of interest	No evidence of spin bias	This is reported in the methodology report but not in the main paper.
7	Conclusion focusing selectively on statistically significant efficacy outcome	No evidence of spin bias	NA
8	Selective reporting of analysis favoring the beneficial effect of the experimental intervention (e.g., selective analysis using random or fixed effect according to the results)	NA	The main paper only provides a narrative synthesis of all included studies, regardless of whether they contain statistically significant results.
9	Inadequate focus on the results of primary studies favoring the beneficial effect of the experimental intervention instead of the meta-analysis results	NA	The main paper provides a narrative synthesis of the results of included studies. No meta-analysis was conducted.
10	Changing the scale of the forest plot to magnify the results (diamond size)	NA	Results were not presented using forest plots.
Misleading interpretation			
11	Title claims or suggests a beneficial effect of the experimental intervention not supported by the findings	No evidence of spin bias	NA
12	Inadequate interpretation of nonstatistically significant results (with a wide confidence interval) as a lack of effect or an equivalent effect for efficacy outcomes	No evidence of spin bias	NA
13	Inadequate interpretation of nonstatistically significant results (with a wide confidence interval) as demonstrating safety for harm outcome	NA	No safety outcomes were assessed.
14	Inadequate focus on *P* value instead of magnitude of the effect estimates for harm or efficacy outcome	No evidence of spin bias	Although the results are narratively synthesized depending on statistical significance, estimates of the magnitude of effects are provided in the summary table. There is no disproportionate focus on *P* values alone.
15	Focus on relative effect when the absolute effect is small	NA	The main paper provides a narrative synthesis of the results of included studies. No meta-analysis was conducted.
16	Misleading interpretation of cited articles, favoring the beneficial effect of the experimental intervention	No evidence of spin bias	NA
17	Conclusion claiming equivalence or comparable effectiveness for nonstatistically significant results with a wide confidence interval	No evidence of spin bias	NA
18	Conclusion formulating recommendations for clinical practice not supported by the findings	No evidence of spin bias	NA
19	Conclusion claiming safety based on nonstatistically significant results with a wide confidence interval	NA	No safety outcomes were assessed.
20	Conclusion ignoring the high risk of bias of the studies, the heterogeneity, or the reporting bias (i.e., a low level of evidence) in the interpretation of the results	No evidence of spin bias	The conclusion statement is general and does not draw any inferences. The overall certainty of evidence is acknowledged to be low, although the potential impact of risk of bias, reporting bias, or heterogeneity in the included studies is not explicitly mentioned. Risk of bias assessments show serious concerns for confounders and moderate concern for selective reporting.
21	No or inadequate consideration of heterogeneity in results interpretation (i.e., no assessment of heterogeneity reported, claiming the absence of heterogeneity not supported by the data, claiming the beneficial effect of the treatment despite high heterogeneity, no downgrading the evidence in cases of high heterogeneity, interpreting nonstatistical significant results for the test of heterogeneity as evidence of no heterogeneity, etc.)	No evidence of spin bias	Because no meta-analyses were performed, statistical heterogeneity was not assessed. The main manuscript discussed heterogeneity between studies and mentions the overall limitations of the evidence.
22	No or inadequate consideration of the risk of bias of primary studies included in results interpretation (i.e., no reporting of the risk of bias of the primary studies, claiming the low risk of bias of studies included not supported by the data, no downgrading the evidence despite several high risk of bias studies)	No evidence of spin bias	NA
Inappropriate extrapolation			
23	No or inadequate consideration of reporting bias in results interpretation (i.e., no reporting of an assessment of reporting bias, claiming efficacy despite evidence of reporting bias, claiming the absence of reporting bias not supported by the data, negative test result interpreted as absence of publication bias, use of the test without the condition of validity, inadequate interpretation of a funnel plot, etc.)	Not enough information to judge	The main manuscript does not report any information on reporting and publication bias. The authors do not claim efficacy despite the absence of reporting and publication bias assessment.
24	Inadequate extrapolation of the results from surrogate markers or specific outcome to the global improvement of the disease	No evidence of spin bias	NA
25	Inadequate extrapolation of the results to a larger population, a larger setting, or a wider set of interventions (e.g., from a specific rehabilitation program to all rehabilitation programs, to a specialized unit to a nonspecialized medical unit, etc.)	No evidence of spin bias	NA
26	Conclusion extrapolating the SR’s findings to a different population or setting	No evidence of spin bias	Although the majority of included studies were conducted in Western countries, the authors do not extrapolate findings outside the United States population.
27	Conclusion extrapolating the SR’s findings to a different intervention (i.e., claiming efficacy of one specific intervention although the SR covers a class of several interventions)	No evidence of spin bias	NA
28	Conclusion extrapolating the SR’s findings from a surrogate marker or a specific outcome to the global improvement of the disease	No evidence of spin bias	NA

Abbreviations: NA, not applicable; NESR, Nutrition Evidence Systematic Review; RCT, randomized controlled trial; SR, systematic review.

#### Data analysis

For KQ2, we aimed to evaluate if any observed differences from reproducing the search strategy and data analysis would change the original conclusions made by the NESR team. Several errors and inconsistencies were identified in the search strategy reported by the authors, which also could not be reproduced within 10% of the original results. In line with PRISMA 2020 assessment results, the assessment of the reporting transparency of the narrative data synthesis using the SWiM checklist identified major concerns, with only 1 of 9 items entirely reported. Separation of studies using the PICOT framework, as well as the meta-analyses that were conducted in the present reproducibility study, identified high levels of clinical and statistical heterogeneity, confirming that using a quantitative synthesis approach was not suited for the NESR team’s SR. Although our assessments suggest that meta-analysis would not have been a suitable approach for analyzing and summarizing results, the possibility of conducting meta-analyses should have been considered by the authors, instead of making the a priori decision of narratively synthesizing the results without proper justification. The meta-analyses further confirmed that, despite differences in study characteristics and exposure measures, the results consistently indicated a positive association between healthier dietary patterns and improved neurocognitive outcomes, which aligns with the NESR team’s narrative summaries. Besides insufficient reporting on some components of the results and the discussion, spin bias assessment revealed no significant problems with the interpretation of results.

## Discussion

### Summary of results

The present study assessed the reliability and reproducibility of selected SRs informing the 2020–2025 DGA using various tools and attempting to reproduce components of one SR. Concerns were identified, particularly in the synthesis and analysis methods, reporting, and search strategy. Despite concerns about narrative data synthesis reporting transparency, meta-analyses confirmed it was appropriate due to significant heterogeneity, and no interpretation bias was observed. Addressing some of these issues could potentially alter the final findings of the selected SRs, although to what extent remains unclear.

### Findings in relation to other studies

All SRs included in this study were of critically low quality based on the AMSTAR 2 assessment, and the reporting transparency of the SRs, and particularly their search strategies, was also deemed suboptimal. Overall, these findings align with those of previous studies that have also applied such assessment tools to evidence syntheses. A study examining SRs underpinning pediatric content in United States CPGs reported overall poor methodological quality and reporting transparency, with items related to risk of bias assessment and search strategies having especially low scores [56]. Along the same lines, 3 recent studies assessing the methodological quality of SRs underlying CPGs in the fields of cardiology and oncology reported a high prevalence of critically low-quality SRs, with numbers ranging from 40% to 60% [[57], [58], [59]]. The methodological quality and reporting transparency assessments performed for KQ1 pointed to difficulties with data synthesis and analysis methods, which has not been identified by previous studies [56,57,60,61]. Interestingly, a study assessing factors associated with the methodological quality of SRs reported that performing a meta-analysis was associated with significantly higher methodological quality as assessed with the AMSTAR 2 tool [62]. Therefore, it is possible that the a priori decision of not performing a meta-analysis negatively impacted the overall methodological quality of the NESR team’s SRs. Regarding the reporting transparency of the search strategy, a previous study assessing SRs on the role of telehealth during the COVID-19 pandemic found that >50% did not provide a full search strategy, which was reflected in the number of adequately fulfilled PRISMA-S checklist items, averaging 6 of 16 items [63]. Although still suboptimal, PRISMA-S scores were higher in this study, with 10 of the 16 PRISMA-S checklist items satisfactorily fulfilled.

When attempting to reproduce the SR search strategy, results differed from those reported in the original SR, indicating failure to reproduce the search strategy within a 10% margin of the original SR results. Previous studies have reported poor reproducibility and reporting of SRs’ search strategies. Despite the increased availability of SR reporting tools such as PRISMA 2020 and PRISMA-S, these issues have also persisted in recent SRs published in 2018 and 2021 [43,64]. The analysis by Rethlefsen et al. [43] of 453 database searches identified in a sample of 100 SRs indicated that as few as 47 (10.4%) searches could be reproduced within 10% of the original number of results, with the most commonly occurring errors being Boolean logic errors, such as phrasing or missing parentheses. Boolean logic errors, such as lack of quotation marks when searching multiword terms, were also noted in this study when the search strategy of the selected SR was evaluated using the PRESS checklist.

In this study, adherence to SWiM reporting guidelines was poor, with only 1 of 9 items being completely reported by the NESR team. Although there are currently no studies assessing the level of adherence to the SWiM reporting guidelines of SRs without meta-analysis, there have been reports of poor reproducibility of meta-analysis results [65,66]. Regarding the interpretation of the results of the selected NESR team’s SR, our spin bias assessment revealed no evidence of misleading reporting, misleading interpretation, and inappropriate extrapolation in both the main text and the abstract. In contrast with our findings, previous studies have identified spin bias in the abstracts of 31% and 74% of SRs conducted in the fields of dermatology and pain management, respectively [67,68].

### Study implications

As the publication rate of SRs continues to increase [43,69], the improvement in SR reporting transparency and reproducibility seems to fall behind, with poorly conducted and reported SRs continuing to be published [[70], [71], [72]]. Given the common use of SRs as high-quality, rigorously conducted, and methodologically sound sources of evidence for the development of CPGs such as the DGA, this raises important concerns for the associated recommendations and their potential impact on the target population and health policy development. The accumulating body of knowledge identifying nonnegligible methodological, reporting, and reproducibility concerns in SRs used to develop CPGs is also susceptible of influencing clinicians’ and public health entities’ perceptions of the quality of CPGs, potentially impacting their implementation. Addressing reproducibility issues in SRs necessitates a comprehensive approach involving various collaborators, such as SR experts, SR authors, information specialists, journal editorial teams, and peer reviewers.

One approach to enhancing SR methodological quality and reproducibility involves mandating prospective protocol registration and the use of reporting guidelines by funding agencies, publishing journals, and CPG developers. Despite having a seemingly well-established, publicly available process for conducting SRs used for the development of the DGA, the NESR team does not report registering SR protocols, following internationally recognized methods (e.g., Cochrane Methods), or using any of the well-established guidance tools (e.g., AMSTAR 2, PRISMA 2020) when planning, conducting, and reporting their SRs. This may be due, in part, to the publication date and slower adoption of more recently released tools such as PRISMA 2020, PRISMA-S, and SWiM. Although the NESR team omitting the use of more recently published guidance tools is understandable, it is evident that nutrition CPGs are likely to be more credible when the inputted SRs are themselves credible in terms of generally accepted methodology and reporting.

Although there is evidence suggesting that publishing journals requiring prospective protocol registration, mandating the use of the PRISMA statement, and reinforcing adherence to well-established methods may enhance the methodological quality and reporting transparency of SRs [[73], [74], [75], [76], [77], [78], [79], [80]], the prevalence of these practices seems relatively low. Among recommended practices, using a protocol is known to improve the detection of reporting biases, whereas protocol registration helps reduce publication bias, research duplication, and research waste [[81], [82], [83]]. Although being suboptimally practiced [84,85], several studies support that protocol registration is associated with significantly higher SR methodological quality and reporting transparency [[73], [74], [75]]. Regarding the association between mandatory adherence to the PRISMA checklist, methodological quality, and reporting transparency, mixed results have been reported by cross-sectional studies [77,79,80]. A study conducted in the field of gastroenterology and hepatology found that the methodological quality and reporting transparency of SRs published in journals endorsing the use of the PRISMA checklist was significantly higher than those published in journals not acknowledging it [77]. Other studies have reported no significant difference in the methodological quality of SRs published in journals with and without PRISMA checklist endorsement [79,80], which may be explained by the fact that endorsement of the PRISMA checklist by publishing journals does not necessarily guarantee that authors will use and adhere to it. These findings reinforce the importance of also offering education on quality assessment tools and reporting guidelines to SR authors, editors, and peer reviewers to improve adherence [86].

A second approach to enhancing SR methodological quality and reproducibility involves including both methodological and content experts in the SR team, such as information specialists and meta-analysis experts, at appropriate stages. Public and patient involvement in several or all stages of SRs may also ensure that they are relevant and meaningful to individuals in the target population while also potentially enhancing relevance, improving quality, and promoting dissemination of findings [87]. Despite these benefits, a recent study including 217 SRs reported that only 56 (26%) of them used public and patient involvement as part of their SR process [88]. The NESR team comprises a variety of SR and methodological experts, such as SR analysts and information specialists as well as content experts, including individuals holding advanced degrees in nutrition, public health, and epidemiology. Additionally, public and patient involvement is apparent at various stages of the SR process, including identifying high-priority SR questions, developing the SR protocol, grading the strength of the evidence, and developing conclusion statements. Interestingly, despite having a well-rounded SR team, the reporting and reproducibility of literature search strategies as well as the reporting of methods used to synthesize and analyze the results of the NESR team’s SR were still largely suboptimal. In this regard, studies have contrarily shown that SRs coauthored by information specialists were more likely to be reproducible and adhere to methodological standards than SRs with no information specialist participation and SRs that mentioned the participation of an information specialist but no coauthorship [[89], [90], [91], [92], [93]]. As SRs with important search strategy and meta-analysis errors are currently still being published following both editorial and peer review, mandatorily including such experts in publishing journals and other editorial teams might also be beneficial.

Finally, a third approach to enhancing SR methodological quality and reproducibility involves promoting open science practices (e.g., registration, open protocols, open materials, open data, open code, open peer review, and open-access publishing). Although they adhere to some open science practices such as open protocol and open-access publishing, the NESR team does not appear to use registration, open materials, open data, open code, or open peer review. Open science practices at all stages of the research lifecycle are well known to individually and collectively enhance research transparency and reproducibility while leveling access to research and fostering accountability and trustworthiness [[94], [95], [96]]. To reflect the evolving expectations arising from the open science movement, the latest version of the PRISMA checklist promotes open science practices by including a new item on the availability of data, analytic code, and other SR materials, along with items on SR protocol and registration that were already included in the previous version of the PRISMA checklist [5,97]. Although the use of some open science practices, such as open-access publishing, has drastically increased over the past 2 decades [94], the use of other open science practices, such as registration, open data, and open code, remain relatively low [98,99]. In addition to promoting or mandating the use of open science practices by acting on common barriers to using them [100], including lack of training and perceived negative impacts of such practices, could be beneficial.

### Strengths and limitations

This study has several strengths. To ensure transparency, consistency, and completeness, the study was conducted following a protocol that was developed and prospectively registered on the OSF platform and that is reported following the PRISMA 2020 checklist. These practices reduce the likelihood of selective outcome reporting, statistical manipulation, and other biased practices. The reliability and reproducibility of the included SRs were assessed using tools that were validated for their content, such as PRISMA 2020, AMSTAR 2, PRISMA-S, and PRESS. To enhance reliability, all assessments were either performed in duplicate or verified by a second member of the research team. Duplicate independent assessment represents the gold standard for SR methodology and is widely supported by Cochrane.

The findings of this study are limited by the fact that we did not attempt to reproduce the entire SR process and restricted the reproducibility exercise to the search strategy and data analysis due to resource constraints. A complete attempt at SR reproduction should also include screening the reproduced literature search results, extracting data, and assessing the risk of bias in individual studies. Further, we did not conduct a thorough exploration of the reasons behind the discrepancy in the volume of records obtained in the replicated literature search. For example, a portion of the observed difference may be explained by the inability to rerun searches on the exact date the original searches were executed. Future research should also assess the quality of evidence and the strength of recommendations provided in the DGA using the Grading of Recommendations Assessment, Development and Evaluation assessment tool. This aspect was beyond the scope of the present study.

Finally, although our study aimed to inform the reliability and reproducibility of all SRs conducted by the NESR team, the generalizability of our results may be limited by our small sample. Our KQ1 evaluated SRs within the largest DGA subcommittee alone (dietary patterns), and there may be differences in SR methodology across the 6 DGA subcommittees’ products. Additionally, due to resource constraints, only one medium-sized SR was selected for partial reproduction to address KQ2. Due to the heterogeneity and the lack of studies within the selected SR, we were only able to estimate statistical heterogeneity for 2 observational outcomes and no randomized controlled trials. Finally, the weaknesses identified in the methodological quality and reporting transparency of the selected SRs may vary across the NESR team’s SRs. This concern may be somewhat mitigated by the fact that all the NESR team’s SRs are informed by the DGA methodology manual and that our KQ1 findings confirmed a high degree of consistency in methodological and reporting transparency across SRs.

## Conclusions

Given the importance of CPGs, ensuring that they are based on high-quality, reproducible SRs is essential. Concerns regarding the methodology, rigor, and transparency of SRs informing the DGA have been continuously expressed over the years [[21], [22], [23], [24], [25],^27^,^28^]. The present study further reinforces that the methodological quality and reporting transparency of SRs informing the 2020–2025 DGA are suboptimal, which could lead to reliability and reproducibility issues. Addressing the methodological concerns identified in the present study could potentially alter the SR findings and the associated DGA. However, additional research is needed to confirm the impact of these issues on the overall conclusions of the NESR team and the generalizability of these findings across the team’s SRs. Studies with larger sample sizes and studies attempting to fully reproduce the NESR team’s SRs are needed.

## Acknowledgments

We thank Dr Brian Hutton for his advice and consultation on meta-analysis. We thank Taddele Kibret for his guidance on statistical analysis.

## Author contributions

The authors’ responsibilities were as follows – NS, AB, BS, JL, MB, DB, DM: designed the research; HK, AMB, NS, AB, YLW: conducted the research; HK, AMB, NS, DM: analyzed the data; HK, AMB, NS: wrote the paper; VL, CG: assessed and attempted to reproduce the search strategy; and all authors: read and approved the final manuscript.

## Funding

This work was supported by a grant from the Nutrition Coalition (https://www.nutritioncoalition.us) to DM. The Nutrition Coalition is a 501(c)(3) nonprofit organization committed to ensuring evidence-based nutrition policies to reverse chronic disease in the United States. Its funding comes exclusively from individuals and charitable organizations, with no contributions accepted from industry sources. The funder had no role in the study design, execution, analyses, interpretation of results, or in the decision to submit the results for publication.

## Data availability

Data described in the manuscript, code book, and analytic code will be made publicly and freely available without restriction at https://osf.io/ns6a9/.

## Conflict of interest

DB was a member of the 1995 DGAC. He has also published an opinion piece on the process [24]. All other authors report no conflicts of interest.
