# Supplementary material for: Reliability and reproducibility of systematic reviews informing the 2020–2025 Dietary Guidelines for Americans: a pilot study
Source: Am J Clin Nutr. 2024 Dec 12;121(1):111–24. doi: 10.1016/j.ajcnut.2024.10.013 (PMC11747194; doi:10.1016/j.ajcnut.2024.10.013)
Supplement: multimedia component 1 [file mmc1.pdf]

Reliability and reproducibility of systematic reviews informing the 2020-2025 Dietary Guidelines for Americans: a pilot study
Bodnaruc et al.

Supplementary file 1. PRISMA 2020 checklist

PRISMA 2020 checklist	Answer (yes/no)	Page Located
**Title**		
1. Title identifies the report as a systematic review.	N/A (Study identifies report as a pilot evaluation evaluating reliability and reproducibility of SRs, p.1)	N/A
**Abstract**		
2. Report an abstract addressing each item in the PRISMA 2020 for abstracts checklist (see Supplementary table 3).	Yes	2
**Introduction**		
3. Describes the rationale for the review in the context of existing knowledge.	Yes	4-5
4. Provides an explicit statement of the objective(s) or question(s) the review addresses.	Yes	6
**Methods**		
5. Specify the inclusion and exclusion criteria for the review and how studies were grouped for the syntheses.	Yes	9-10
6. Specify all databases, registers, websites, organisations, reference lists and other sources searched or consulted to identify studies. They also specify the date when each source was last searched or consulted.	Yes	9; Supplementary file 6
7. Present the full search strategies for all databases, registers and websites, including any filters and limits used.	Yes	9; Supplementary file 6
8. Specify the methods used to decide whether a study met the inclusion criteria of the review, including how many reviewers screened each record and each report retrieved, whether they worked independently, and if applicable, details of automation tools used in the process.	N/A (No reproduction of screening)	N/A
9. Specify the methods used to collect data from reports, including how many reviewers collected data from each report, whether they worked independently, any processes for obtaining or confirming data from study investigators, and if applicable, details of automation tools used in the process.	N/A (No reproduction of data extraction)	N/A

10a. List and define all outcomes for which data were sought. Specify whether all results that were compatible with each outcome domain in each study were sought (e.g. for all measures, time points, analyses), and if not, the methods used to decide which results to collect.	N/A (No reproduction of data extraction)	N/A
10b. List and define all other variables for which data were sought (e.g. participant and intervention characteristics, funding sources). Describe any assumptions made about any missing or unclear information.	N/A (No reproduction of data extraction)	N/A
11. Specify the methods used to assess risk of bias in the included studies, including details of the tool(s) used, how many reviewers assessed each study and whether they worked independently, and if applicable, details of automation tools used in the process.	N/A (No reproduction of risk of bias assessments); Assessment of SRs evaluated via AMSTAR-2	N/A; 7
12. Specify for each outcome the effect measure(s) (e.g. risk ratio, mean difference) used in the synthesis or presentation of results.	Yes	P.10
13a. Describe the processes used to decide which studies were eligible for each synthesis (e.g. tabulating the study intervention characteristics and comparing against the planned groups for each synthesis (item #5)).	Yes	P.10
13b. Describe any methods required to prepare the data for presentation or synthesis, such as handling of missing summary statistics, or data conversions.	N/A	N/A
13c. Describe any methods used to tabulate or visually display results of individual studies and syntheses.	Yes	P.10
13d. Describe any methods used to synthesize results and provide a rationale for the choice(s). If meta-analysis was performed, describe the model(s), method(s) to identify the presence and extent of statistical heterogeneity, and software package(s) used.	Yes	P.10
13e. Describe any methods used to explore possible causes of heterogeneity among study results (e.g. subgroup analysis, meta-regression).	Yes	P.10
13f. Describe any sensitivity analyses conducted to assess robustness of the synthesized results.	N/A	N/A
14. Describe any methods used to assess risk of bias due to missing results in a synthesis (arising from reporting biases).	N/A	N/A
15. Describe any methods used to assess certainty (or confidence) in the body of evidence for an outcome.	N/A	N/A
**Results**		
16a. Describe the results of the search and selection process, from the number of records identified in the search to the number of studies included in the review, ideally using a flow diagram.	Yes (Results of search result reproduction)	14-15; Supplementary file 6
16b. Cite studies that might appear to meet the inclusion criteria, but which were excluded, and explain why they were excluded.	N/A	N/A

17. Cite each included study and present its characteristics.	N/A	N/A
18. Present assessments of risk of bias for each included study.	N/A	N/A
19. For all outcomes, present, for each study: (a) summary statistics for each group (where appropriate) and (b) an effect estimate and its precision (e.g. confidence/credible interval), ideally using structured tables or plots.	Yes	16; Figure 2
20a. For each synthesis, briefly summarise the characteristics and risk of bias among contributing studies.	N/A (No reproduction of RoB Assessments) ; Assessment of SRs evaluated via AMSTAR-2	N/A; 12
20b. Present results of all statistical syntheses conducted. If meta-analysis was done, present for each the summary estimate and its precision (e.g. confidence/credible interval) and measures of statistical heterogeneity. If comparing groups, describe the direction of the effect.	Yes	16; Figure 2
20c. Present results of all investigations of possible causes of heterogeneity among study results.	Yes	15-17
20d. Present results of all sensitivity analyses conducted to assess the robustness of the synthesized results.	N/A	N/A
21. Present assessments of risk of bias due to missing results (arising from reporting biases) for each synthesis assessed.	N/A	N/A
22. Present assessments of certainty (or confidence) in the body of evidence for each outcome assessed.	N/A	N/A
**Discussion**		
23a. Provide a general interpretation of the results in the context of other evidence.	Yes	18-22
23b. Discuss any limitations of the evidence included in the review.	Yes	18-19
23c. Discuss any limitations of the review processes used.	Yes	27-28
23d. Discuss implications of the results for practice, policy, and future research.	Yes	22-27
**Other information**		
24a. Provide registration information for the review, including register name and registration number, or state that the review was not registered.	Yes	3
24b. Indicate where the review protocol can be accessed, or state that a protocol was not prepared.	Yes	6
24c. Describe and explain any amendments to information provided at registration or in the protocol.	Yes	6
25. Describe sources of financial or non-financial support for the review, and the role of the funders or sponsors in the review.	Yes	29
26. Declare any competing interests of review authors.	Yes	29

27. Report which of the following are publicly available and where they can be found: template data collection forms; data extracted from included studies; data used for all analyses; analytic code; any other materials used in the review.	Yes	29

Reliability and reproducibility of systematic reviews informing the 2020-2025 Dietary Guidelines for Americans: a pilot study
Bodnaruc et al.

Supplementary file 2. PRISMA 2020 for Abstracts Checklist

**PRISMA 2020 for abstracts checklist**	**Answer** (yes/no)
**Title**	
1. Identify the report as a systematic review.	N/A
**Background**	
2. Provide an explicit statement of the main objective(s) or question(s) the review addresses.	Yes
Methods	
3. Specify the inclusion and exclusion criteria for the review.	N/A
4. Specify the information sources (e.g. databases, registers) used to identify studies and the date when each was last searched.	N/A
5. Specify the methods used to assess risk of bias in the included studies.	Yes
6. Specify the methods used to present and synthesise results.	Yes
Results	
7. Give the total number of included studies and participants and summarise relevant characteristics of studies.	N/A
8. Present results for main outcomes, preferably indicating the number of included studies and participants for each. If meta-analysis was done, report the summary estimate and confidence/credible interval. If comparing groups, indicate the direction of the effect (i.e. which group is favoured).	N/A
Discussion	
9. Provide a brief summary of the limitations of the evidence included in the review (e.g. study risk of bias, inconsistency and imprecision).	Yes
10. Provide a general interpretation of the results and important implications.	Yes
Other	
11. Specify the primary source of funding for the review.	Yes
12. Provide the register name and registration number.	Yes

Reliability and reproducibility of systematic reviews informing the 2020-2025 Dietary Guidelines for Americans: a pilot study
Bodnaruc et al.

Supplementary file 3. AMSTAR 2 assessment of NESR team's SRs on dietary patterns and health outcomes

Topic	Author	1	2*	3	4*	5	6	7*	8	9*		10	11*		12	13*	14	15*	16	Overall rating
										a	b		a	b						
All-cause mortality	Boushey et al. (2020) [90]	Y	PY	Y	Y	Y	Y	Y	PY	Y	Y	Y	N	N	NA	Y	N	NA	Y	Critically low
Bone health	Boushey et al. (2020) [91]	Y	PY	Y	PY	Y	Y	Y	PY	Y	Y	N	N	N	NA	Y	N	NA	Y	Critically low
Cancer	Boushey et al. (2020) [92]	Y	PY	Y	PY	Y	Y	Y	PY	Y	Y	Y	N	N	NA	Y	N	NA	Y	Critically low
Cardiovascular disease	Boushey et al. (2020) [93]	Y	PY	Y	Y	Y	Y	Y	Y	Y	Y	Y	N	N	NA	Y	N	NA	Y	Critically low
Obesity	Boushey et al. (2020) [94]	Y	PY	Y	Y	Y	Y	Y	Y	Y	Y	Y	N	N	NA	Y	N	NA	Y	Critically low
Neurocognitive health	Boushey et al. (2020) [34]	Y	PY	Y	Y	Y	Y	Y	Y	Y	Y	Y	N	N	NA	Y	N	NA	Y	Critically low
Sarcopenia	Boushey et al. (2020) [95]	Y	PY	Y	PY	Y	Y	Y	Y	Y	Y	Y	N	N	NA	Y	N	NA	Y	Critically low
Type 2 diabetes	Boushey et al. (2020) [96]	Y	PY	Y	Y	Y	Y	Y	Y	Y	Y	Y	N	N	NA	Y	N	NA	Y	Critically low

* indicates critical domains
NA: Non-applicable (because no meta-analysis was done); N: No; PY: Partial yes; Y: Yes

Question Key:
1. Did the research questions and inclusion criteria for the review include the components of PICO?
2. Did the report of the review contain an explicit statement that the review methods were established prior to the conduct of the review and did the report justify any significant deviations from the protocol?
3. Did the review authors explain their selection of the study designs for inclusion in the review?
4. Did the review authors use a comprehensive literature search strategy?
5. Did the review authors perform study selection in duplicate?
6. Did the review authors perform data extraction in duplicate?
7. Did the review authors provide a list of excluded studies and justify the exclusions?
8. Did the review authors describe the included studies in adequate detail?
9a. Did the review authors use a satisfactory technique for assessing the risk of bias (RoB) in individual studies that were included in the review? (RCTs)
9b. Did the review authors use a satisfactory technique for assessing the risk of bias (RoB) in individual studies that were included in the review? (NRSIs)
10. Did the review authors report on the sources of funding for the studies included in the review?
11a. If meta-analysis was performed did the review authors use appropriate methods for statistical combination of results? (RCTs)
11b. If meta-analysis was performed did the review authors use appropriate methods for statistical combination of results? (NRSIs)
12. If meta-analysis was performed, did the review authors assess the potential impact of RoB in individual studies on the results of the meta-analysis or other evidence synthesis?
13. Did the review authors account for RoB in individual studies when interpreting/ discussing the results of the review?
14. Did the review authors provide a satisfactory explanation for, and discussion of, any heterogeneity observed in the results of the review?
15. If they performed quantitative synthesis did the review authors carry out an adequate investigation of publication bias (small study bias) and discuss its likely impact on the results of the review?
16. Did the review authors report any potential sources of conflict of interest, including any funding they received for conducting the review?

Citation: Shea BJ, Reeves BC, Wells G, Thuku M, Hamel C, Moran J, Moher D, Tugwell P, Welch V, Kristjansson E, Henry DA. AMSTAR 2: a critical appraisal tool for systematic reviews that include randomised or non-randomised studies of healthcare interventions, or both. BMJ. 2017 Sep 21;358:j4008.

Reliability and reproducibility of systematic reviews informing the 2020-2025 Dietary Guidelines for Americans: a pilot study
Bodnaruc et al.

Supplementary file 4. PRISMA 2020 checklist for NESR team's SRs on dietary patterns and health outcomes

Section and Topic	Item #	Checklist item	Is this item reported? (yes/no) (https://nesr.usda.gov/2020-dietary-guidelines-advisory-committee-systematic-reviews/dietary-patterns-subcommittee)							
			All-cause mortality	Bone health	Cancer	CVD	Obesity	Neurocognitive health	Sarcopenia	Diabetes
**TITLE**										
Title	1	Identify the report as a systematic review.	1	1	1	1	1	1	1	1
**ABSTRACT**										
Abstract	2	See the PRISMA 2020 for Abstracts checklist.	0	0	0	0	0	0	0	0
**INTRODUCTION**										
Rationale	3	Describe the rationale for the review in the context of existing knowledge.	0	0	0	0	0	0	0	0
Objectives	4	Provide an explicit statement of the objective(s) or question(s) the review addresses.	1	1	1	1	1	1	1	1
**METHODS**										
Eligibility criteria	5	Specify the inclusion and exclusion criteria for the review and how studies were grouped for the syntheses.	1	1	1	1	1	1	1	1
Information sources	6	Specify all databases, registers, websites, organisations, reference lists and other sources searched or consulted to identify studies. Specify the date when each source was last searched or consulted.	1	1	1	1	1	1	1	1
Search strategy	7	Present the full search strategies for all databases, registers and websites, including any filters and limits used.	1	1	1	1	1	1	1	1
Selection process	8	Specify the methods used to decide whether a study met the inclusion criteria of the review, including how many reviewers screened each record and each report retrieved, whether they worked independently, and if applicable, details of automation tools used in the process.	1	1	1	1	1	1	1	1
Data collection process	9	Specify the methods used to collect data from reports, including how many reviewers collected data from each report, whether they worked independently, any processes for obtaining or confirming data from study investigators, and if applicable, details of automation tools used in the process.	1	1	1	1	1	1	1	1
Data items	10a	List and define all outcomes for which data were sought. Specify whether all results that were compatible with each outcome domain in each study were sought (e.g. for all measures, time points, analyses), and if not, the methods used to decide which results to collect.	1	1	1	1	1	1	1	1

	10b	List and define all other variables for which data were sought (e.g. participant and intervention characteristics, funding sources). Describe any assumptions made about any missing or unclear information.	1	1	1	1	1	1	1	1
Study risk of bias assessment	11	Specify the methods used to assess risk of bias in the included studies, including details of the tool(s) used, how many reviewers assessed each study and whether they worked independently, and if applicable, details of automation tools used in the process.	1	1	1	1	1	1	1	1
Effect measures	12	Specify for each outcome the effect measure(s) (e.g. risk ratio, mean difference) used in the synthesis or presentation of results.	1	1	1	1	1	1	1	1
Synthesis methods	13a	Describe the processes used to decide which studies were eligible for each synthesis (e.g. tabulating the study intervention characteristics and comparing against the planned groups for each synthesis (item #5)).	1	1	1	1	1	1	1	1
	13b	Describe any methods required to prepare the data for presentation or synthesis, such as handling of missing summary statistics, or data conversions.	0	0	0	0	0	0	0	0
	13c	Describe any methods used to tabulate or visually display results of individual studies and syntheses.	1	1	1	1	1	1	1	1
	13d	Describe any methods used to synthesize results and provide a rationale for the choice(s). If meta-analysis was performed, describe the model(s), method(s) to identify the presence and extent of statistical heterogeneity, and software package(s) used.	0	0	0	0	0	0	0	0
	13e	Describe any methods used to explore possible causes of heterogeneity among study results (e.g. subgroup analysis, meta-regression).	0	0	0	0	0	0	0	0
	13f	Describe any sensitivity analyses conducted to assess robustness of the synthesized results.	0	0	0	0	0	0	0	0
Reporting bias assessment	14	Describe any methods used to assess risk of bias due to missing results in a synthesis (arising from reporting biases).	1	1	1	1	1	1	1	1
Certainty assessment	15	Describe any methods used to assess certainty (or confidence) in the body of evidence for an outcome.	1	1	1	1	1	1	1	1
**RESULTS**										
Study selection	16a	Describe the results of the search and selection process, from the number of records identified in the search to the number of studies included in the review, ideally using a flow diagram.	1	1	1	1	1	1	1	1
	16b	Cite studies that might appear to meet the inclusion criteria, but which were excluded, and explain why they were excluded.	1	1	1	1	1	1	1	1
Study characteristics	17	Cite each included study and present its characteristics.	1	1	1	1	1	1	1	1
Risk of bias in studies	18	Present assessments of risk of bias for each included study.	1	1	1	1	1	1	1	1
Results of individual studies	19	For all outcomes, present, for each study: (a) summary statistics for each group (where appropriate) and (b) an effect estimate and its precision (e.g. confidence/credible interval), ideally using structured tables or plots.	1	1	1	1	1	1	1	1

Results of syntheses	20a	For each synthesis, briefly summarise the characteristics and risk of bias among contributing studies.	1	1	1	1	1	1	1	1
	20b	Present results of all statistical syntheses conducted. If meta-analysis was done, present for each the summary estimate and its precision (e.g. confidence/credible interval) and measures of statistical heterogeneity. If comparing groups, describe the direction of the effect.	0	0	0	0	0	0	0	0
	20c	Present results of all investigations of possible causes of heterogeneity among study results.	0	0	0	0	0	0	0	0
	20d	Present results of all sensitivity analyses conducted to assess the robustness of the synthesized results.	0	0	0	0	0	0	0	0
Reporting biases	21	Present assessments of risk of bias due to missing results (arising from reporting biases) for each synthesis assessed.	1	1	1	1	1	1	1	1
Certainty of evidence	22	Present assessments of certainty (or confidence) in the body of evidence for each outcome assessed.	1	1	1	1	1	1	1	1
**DISCUSSION**										
Discussion	23a	Provide a general interpretation of the results in the context of other evidence.	0	0	0	0	0	0	0	0
	23b	Discuss any limitations of the evidence included in the review.	1	1	1	1	1	1	1	1
	23c	Discuss any limitations of the review processes used.	0	0	0	0	0	0	0	0
	23d	Discuss implications of the results for practice, policy, and future research.	1	1	1	1	1	1	1	1
**OTHER INFORMATION**										
Registration and protocol	24a	Provide registration information for the review, including register name and registration number, or state that the review was not registered.	0	0	0	0	0	0	0	0
	24b	Indicate where the review protocol can be accessed, or state that a protocol was not prepared.	1	1	1	1	1	1	1	1
	24c	Describe and explain any amendments to information provided at registration or in the protocol.	0	0	0	0	0	0	0	0
Support	25	Describe sources of financial or non-financial support for the review, and the role of the funders or sponsors in the review.	1	1	1	1	1	1	1	1
Competing interests	26	Declare any competing interests of review authors.	1	1	1	1	1	1	1	1
Availability of data, code and other materials	27	Report which of the following are publicly available and where they can be found: template data collection forms; data extracted from included studies; data used for all analyses; analytic code; any other materials used in the review.	0	0	0	0	0	0	0	0

Citation: Page MJ, McKenzie JE, Bossuyt PM, Boutron I, Hoffmann TC, Mulrow CD, et al. The PRISMA 2020 statement: an updated guideline for reporting systematic reviews. BMJ 2021;372:n71. doi: 10.1136/bmj.n71

Reliability and reproducibility of systematic reviews informing the 2020-2025 Dietary Guidelines for Americans: a pilot study
Bodnaruc et al.

Supplementary file 5. PRISMA-S checklist for NESR team's SRs on dietary patterns and health outcomes

Section/topic	#	Checklist item	Location(s) Reported							
			All-cause mortality	Bone health	Cancer	CVD	Obesity	Neurocognitive health	Sarcopenia	Diabetes
**INFORMATION SOURCES AND METHODS**										
Database name	1	Name each individual database searched, stating the platform for each.	Methods (pgs. 237-239)	Methods (pg. 32-38)	Methods (pg. 217-222)	Methods (pg. 91-99)	Methods (pg. 76-81)	Methods (pg. 53-56)	Methods (pg. 33-36)	Methods (pg. 51-59)
Multi-database searching	2	If databases were searched simultaneously on a single platform, state the name of the platform, listing all of the databases searched.	N/A	N/A	N/A	N/A	N/A	N/A	N/A	N/A
Study registries	3	List any study registries searched.	Methods (pgs.237-240)	Methods (pgs. 32-38)	Methods (pg. 217-222)	Methods (pg. 91-99)	Methods (pg. 76-81)	Methods (pg. 53-56)	Methods (pg. 33-36)	Methods (pg. 51-59)
Online resources and browsing	4	Describe any online or print source purposefully searched or browsed (e.g., tables of contents, print conference proceedings, web sites), and how this was done.	Not reported	Not reported	Not reported	Not reported	Not reported	Not reported	Not reported	Not reported
Citation searching	5	Indicate whether cited references or citing references were examined, and describe any methods used for locating cited/citing references (e.g., browsing reference lists, using a citation index, setting up email alerts for references citing included studies).	Methodology (pg. 13)	Methodology (pg. 13)	Methodology (pg. 13)	Methodology (pg. 13)	Methodology (pg. 13)	Methodology (pg. 13)	Methodology (pg. 13)	Methodology (pg. 13)
Contacts	6	Indicate whether additional studies or data were sought by contacting authors, experts, manufacturers, or others.	yes	yes	yes	yes	yes	yes	yes	yes
Other methods	7	Describe any additional information sources or search methods used.	Methodology (pg. 11)	Methodology (pg. 10)	Methodology (pg. 13-14)	Methodology (pg. 14)	Methodology (pg. 13)	Methodology (pg. 11)	Methodology (pg. 10)	Methodology (pg. 10)
**SEARCH STRATEGIES**										
Full search strategies	8	Include the search strategies for each database and information source, copied and pasted exactly as run.	Methods (pgs. 237-240)	Methods (pgs. 32-38)	Methods (pgs. 217-222)	Methods (pgs. 91-99)	Methods (pgs. 76-81)	Methods (pgs. 53-56)	Methods (pgs. 33-36)	Methods (pgs. 51-59)

Limits and restrictions	9	Specify that no limits were used, or describe any limits or restrictions applied to a search (e.g., date or time period, language, study design) and provide justification for their use.	Methodology (pgs. 7-9)	Methodology (pgs. 7-9)	Methodology (pgs. 7-9)	Methodology (pgs. 7-9)	Methodology (pgs. 7-9)	Methodology (pgs. 7-9)	Methodology (pgs. 7-9)	Methodology (pgs. 7-9)
Search filters	10	Indicate whether published search filters were used (as originally designed or modified), and if so, cite the filter(s) used.	Not reported	Not reported	Not reported	Not reported	Not reported	Not reported	Not reported	Not reported
Prior work	11	Indicate when search strategies from other literature reviews were adapted or reused for a substantive part or all of the search, citing the previous review(s).	Not reported	Not reported	Not reported	Not reported	Not reported	Not reported	Not reported	Not reported
Updates	12	Report the methods used to update the search(es) (e.g., rerunning searches, email alerts).	Not reported	Not reported	Not reported	Not reported	Not reported	Not reported	Not reported	Not reported
Dates of searches	13	For each search strategy, provide the date when the last search occurred.	Methods (pgs. 237-240)	Methods (pgs. 32-38)	Methods (pgs. 217-222)	Methods (pgs. 91-99)	Methods (pgs. 76-81)	Methods (pgs. 53-56)	Methods (pgs. 33-36)	Methods (pgs. 51-59)
**PEER REVIEW**										
Peer review	14	Describe any search peer review process.	Methodology (pgs. 10-11)	Methodology (pgs. 10-11)	Methodology (pgs. 10-11)	Methodology (pgs. 10-11)	Methodology (pgs. 10-11)	Methodology (pgs. 10-11)	Methodology (pgs. 10-11)	Methodology (pgs. 10-11)
**MANAGING RECORDS**										
Total Records	15	Document the total number of records identified from each database and other information sources.	Not reported	Not reported	Not reported	Not reported	Not reported	Not reported	Not reported	Not reported
Deduplication	16	Describe the processes and any software used to deduplicate records from multiple database searches and other information sources.	yes	yes	yes	yes	yes	yes	yes	yes

Note: The term 'methodology' refers to the published methodology for conducting reviews, while 'methods' refers to the methods section for that specific review.

Citation: Rethlefsen, M.L.; Kirtley, S.; Waffenschmidt, S.; Ayala, A.P.; Moher, D.; Page, M.J.; Koffel, J.B.; Blunt, H.; Brigham, T.; Chang, S.; et al. PRISMA-S: An Extension to the PRISMA Statement for Reporting Literature Searches in Systematic Reviews. *Syst Rev* 2021, 10, 39.

Reliability and reproducibility of systematic reviews informing the 2020-2025 Dietary Guidelines for Americans: a pilot study
Bodnaruc et al.

Supplementary file 6. Research Replication Results

Search results run by Catherine Gracey, BSc, MI student (Dalhousie University) and Valentina Ly, BSc, MLIS (University of Ottawa)

Search Strategy Run:

Embase

Provider: Elsevier
Date run: August 3, 2023

#	Query	Results
1	'feeding behavior'/de OR 'mediterranean diet'/exp OR 'dash diet'/exp OR 'gluten free diet'/exp OR 'paleolithic diet'/de OR 'vegetarian diet'/exp OR 'healthy diet'/exp OR 'western diet'/de OR 'low carbohydrate diet'/exp OR 'low fat diet'/de OR 'lipid diet'/exp OR 'protein diet'/exp OR 'protein restriction'/exp OR 'sodium restriction'/exp	244,831
2	'dietary pattern*':ab,ti OR 'diet pattern*':ab,ti OR 'eating pattern*':ab,ti OR 'food pattern*':ab,ti OR 'diet quality*':ab,ti OR 'eating habit*':ab,ti OR 'dietary habit*':ab,ti OR 'diet habit*':ab,ti OR 'food habit*':ab,ti OR 'beverage habit*':ab,ti OR 'feeding behavior*':ab,ti OR 'dietary profile*':ab,ti OR 'food profile*':ab,ti OR 'diet profile*':ab,ti OR 'eating profile*':ab,ti OR 'dietary guideline*':ab,ti OR 'dietary recommendation*':ab,ti OR 'dietary intake*':ab,ti OR 'eating style*':ab,ti OR 'mediterranean diet*':ab,ti OR 'dietary approaches to stop hypertension diet*':ab,ti OR 'dash diet*':ab,ti OR 'gluten free diet*':ab,ti OR 'prudent diet*':ab,ti OR 'paleolithic diet*':ab,ti OR 'vegetarian diet*':ab,ti OR 'vegan diet*':ab,ti OR 'healthy diet':ab,ti OR 'plant based diet*':ab,ti OR 'western diet*':ab,ti OR 'low-carbohydrate diet*':ab,ti OR 'high carbohydrate diet*':ab,ti OR 'ketogenic diet*':ab,ti OR 'nordic diet*':ab,ti OR 'high protein diet*':ab,ti OR 'protein intake*':ab,ti OR 'high-fat diet*':ab,ti OR 'low fat diet*':ab,ti OR 'low protein diet*':ab,ti OR 'low-sodium diet*':ab,ti OR 'low salt diet*':ab,ti	180,741
3	('guideline adherence*' NEAR/6 (diet OR dietary OR food OR beverage* OR nutrition*)):ab,ti	40
4	'diet score*':ab,ti OR 'diet quality score*':ab,ti OR 'diet quality index*':ab,ti OR kidmed:ab,ti OR 'diet index*':ab,ti OR 'dietary index*':ab,ti OR 'food score*':ab,ti OR meddietscore:ab,ti OR 'healthy eating index*':ab,ti	7,255

5	((pattern OR patterns OR consumption OR habit*) NEAR/6 (diet OR diets OR dietary OR food OR foods OR beverage OR beverages)):ab,ti	117,069
6	#1 OR #2 OR #3 OR #4 OR #5	415,098
7	'cognitive defect'/exp OR 'cognition'/exp OR 'dementia'/exp	3,467,261
8	cognition:ab,ti OR metacognition:ab,ti OR neurocognitive:ab,ti OR dementia:ab,ti OR alzheimer*:ab,ti OR senility:ab,ti OR senile:ab,ti OR presenile:ab,ti	522,296
9	(cognit* NEAR/6 (function* OR dysfunction* OR declin* OR deteriorat* OR degenerat* OR disorder* OR dysfunction* OR reduct* OR impair* OR deficit* OR deficien* OR progress* OR perform* OR abilit*)):ab,ti	408,342
10	#7 OR #8 OR #9	3,596,771
11	#6 AND #10	44,248
12	#6 AND #10 AND ([article]/lim OR [article in press]/lim) AND [humans]/lim AND [english]/lim AND [2014-2020]/py NOT ([conference abstract]/lim OR [conference paper]/lim OR [conference review]/lim OR [editorial]/lim OR [erratum]/lim OR [letter]/lim OR [note]/lim OR [review]/lim OR [systematic review]/lim OR [meta analysis]/lim)	6,768

PubMed

Provider: U.S. National Library of Medicine
Date run: August 3, 2023

#	Query	Results
	("dietary pattern*" OR "diet pattern*" OR "eating pattern*" OR "food pattern*" OR "diet quality*" OR "eating habit*" OR "dietary habit*" OR "diet habit*" OR "food habit*" OR "beverage habit*" OR "Feeding Behavior"[Mesh:NoExp] OR feeding behavior*[tiab] OR "dietary profile*" OR "food profile*" OR "diet profile*" OR "eating profile*" OR "dietary guideline*" OR "dietary recommendation*" OR "dietary intake*" OR "eating style*" OR "Diet, Mediterranean"[Mesh] OR Mediterranean Diet*[tiab] OR "Dietary Approaches To Stop Hypertension"[Mesh] OR "Dietary Approaches To Stop Hypertension Diet*" OR "DASH diet*" OR "Diet, Gluten-Free"[Mesh] OR "Gluten Free diet*" OR "prudent diet*" OR "Diet, Paleolithic"[Mesh] OR "Paleolithic Diet*"	471,401

	OR "Diet, Vegetarian"[Mesh] OR vegetarian diet*[tiab] OR "vegan diet*" OR "Diet, Healthy"[Mesh] OR "healthy diet*" OR "plant based diet*" OR "Diet, Western"[Mesh] OR "western diet*" OR "Diet, Carbohydrate-Restricted"[Mesh] OR "low-carbohydrate diet*" OR "high carbohydrate diet*" OR "Ketogenic Diet*" OR "Nordic Diet*" OR "Diet, Fat-Restricted"[Mesh] OR "Diet, High-Fat"[Mesh] OR "Diet, High-Protein"[Mesh] OR high protein diet*[tiab] OR "protein intake*" OR "high-fat diet*" OR "low fat diet*" OR "Diet, Protein-Restricted"[Mesh] OR "low protein diet*" OR "Diet, Sodium-Restricted"[Mesh] OR "low-sodium diet*" OR "low salt diet*" OR (("Guideline Adherence"[Mesh] OR "guideline adherence*") AND (diet[tiab] OR dietary[tiab] OR food[tiab] OR beverage*[tiab] OR nutrition*[tiab])) OR "diet score*" OR "diet quality score*" OR "diet quality index*" OR kidmed OR "diet index*" OR "dietary index*" OR "food score*" OR MedDietScore OR healthy eating index[tiab] OR ((pattern[tiab] OR patterns[tiab] OR consumption[tiab] OR habit*[tiab]) AND ("Diet"[Mesh:NoExp] OR diet[tiab] OR diets[tiab] OR dietary[tiab] OR "Food"[Mesh] OR food[tiab] OR foods[tiab] OR "Beverages"[Mesh] OR beverage[tiab] OR beverages[tiab])))	
	("Cognition Disorders"[Mesh] OR "Cognition"[Mesh] OR cognition[tiab] OR metacognition[tiab] OR neurocognitive[tiab] OR "Dementia"[Mesh] OR dementia[tiab] OR Alzheimer*[tiab] OR senility[tiab] OR senile[tiab] OR presenile[tiab] OR (cognit*[tiab] AND (function*[tiab] OR dysfunction*[tiab] OR declin*[tiab] OR deteriorat* OR degenerat*[tiab] OR disorder*[tiab] OR dysfunction*[tiab] OR reduct*[tiab] OR impair*[tiab] OR deficit*[tiab] OR deficien* OR progress*[tiab] OR perform*[tiab] OR abilit*[tiab])))	878,463
	#1 AND #2	13,450
	(#1 AND #2) NOT ("Animals"[Mesh] NOT ("Animals"[Mesh] AND "Humans"[Mesh])) NOT (editorial[ptyp] OR comment[ptyp] OR news[ptyp] OR letter[ptyp] OR review[ptyp] OR systematic review[ptyp] OR systematic review[ti] OR meta-analysis[ptyp] OR meta-analysis[ti] OR meta-analyses[ti] OR retracted publication[ptyp] OR retraction of publication[ptyp] OR retraction of publication[tiab] OR retraction notice[ti]) Filters: English, from 2014/1/1 - 2020/2/4	2,431

Cochrane Central Register of Controlled Trials (CENTRAL)

Provider: John Wiley & Sons
Date Searched: August 4, 2023

#	Query	Results
1	[mh ^"Feeding Behavior"] OR [mh "Diet, Mediterranean"] OR [mh "Dietary Approaches To Stop Hypertension"] OR [mh "Diet, Gluten-Free"] OR [mh "Diet, Paleolithic"] OR [mh "Diet, Vegetarian"] OR [mh "Diet, Healthy"] OR [mh "Diet, Healthy"] OR [mh "Diet, Carbohydrate-Restricted"] OR [mh "Diet, Fat-Restricted"] OR [mh "Diet, High-Fat"] OR [mh "Diet, High-Protein"] OR [mh "Diet, Protein-Restricted"] OR [mh "Diet, Sodium-Restricted"]	8,546
2	("dietary pattern*" OR "diet pattern*" OR "eating pattern*" OR "food pattern*" OR "diet quality*" OR "eating habit*" OR "dietary habit*" OR "diet habit*" OR "food habit*" OR "beverage habit*" OR "feeding behavior*" OR "dietary profile*" OR "food profile*" OR "diet profile*" OR "eating profile*" OR "dietary guideline*" OR "dietary recommendation*" OR "dietary intake*" OR "eating style*" OR "Mediterranean Diet*" OR "Dietary Approaches To Stop Hypertension Diet*" OR "DASH diet*" OR "Gluten Free diet*" OR "prudent diet*" OR "Paleolithic Diet*" OR "vegetarian diet*" OR "vegan diet*" OR "healthy diet*" OR "plant based diet*" OR "western diet*" OR "low-carbohydrate diet*" OR "high carbohydrate diet*" OR "Ketogenic Diet*" OR "Nordic Diet*" OR "high protein diet*" OR "protein intake*" OR "high-fat diet*" OR "low fat diet*" OR "low protein diet*" OR "low-sodium diet*" OR "low salt diet*"):ti,ab,kw	27,287
3	(([mh "Guideline Adherence"] OR guideline adherence*) NEAR/6 (diet OR dietary OR food OR beverage* OR nutrition*))	3,528
4	("diet score*" OR "diet quality score*" OR "diet quality index*" OR kidmed OR "diet index*" OR "dietary index*" OR "food score*" OR MedDietScore OR "healthy eating index*"):ti,ab,kw	758
5	((pattern OR patterns OR consumption OR habit*) NEAR/6 ([mh ^"Diet"] OR diet OR diets OR dietary OR [mh "Food"] OR food OR foods OR [mh "Beverages"] OR beverage OR beverages))	13,630
6	#1 OR #2 OR #3 OR #4 OR #5	38,979

7	[mh "Cognition Disorders"] OR [mh "Cognition"] OR [mh "Dementia"]	28,729
8	(cognition OR metacognition OR neurocognitive OR dementia OR Alzheimer* OR senility OR senile OR presenile):ti,ab,kw	56,068
9	((cognit* NEAR/6 (function* OR dysfunction* OR declin* OR deteriorat* OR degenerat* OR disorder* OR dysfunction* OR reduct* OR impair* OR deficit* OR deficien* OR progress* OR perform* OR abilit*))):ti,ab,kw	51,580
10	#7 OR #8 OR #9	83,255
11	#6 AND #10" with Publication Year from 2014 to 2020, in Trials (Word variations have been searched)	952

Reliability and reproducibility of systematic reviews informing the 2020-2025 Dietary Guidelines for Americans: a pilot study
Bodnaruc et al.

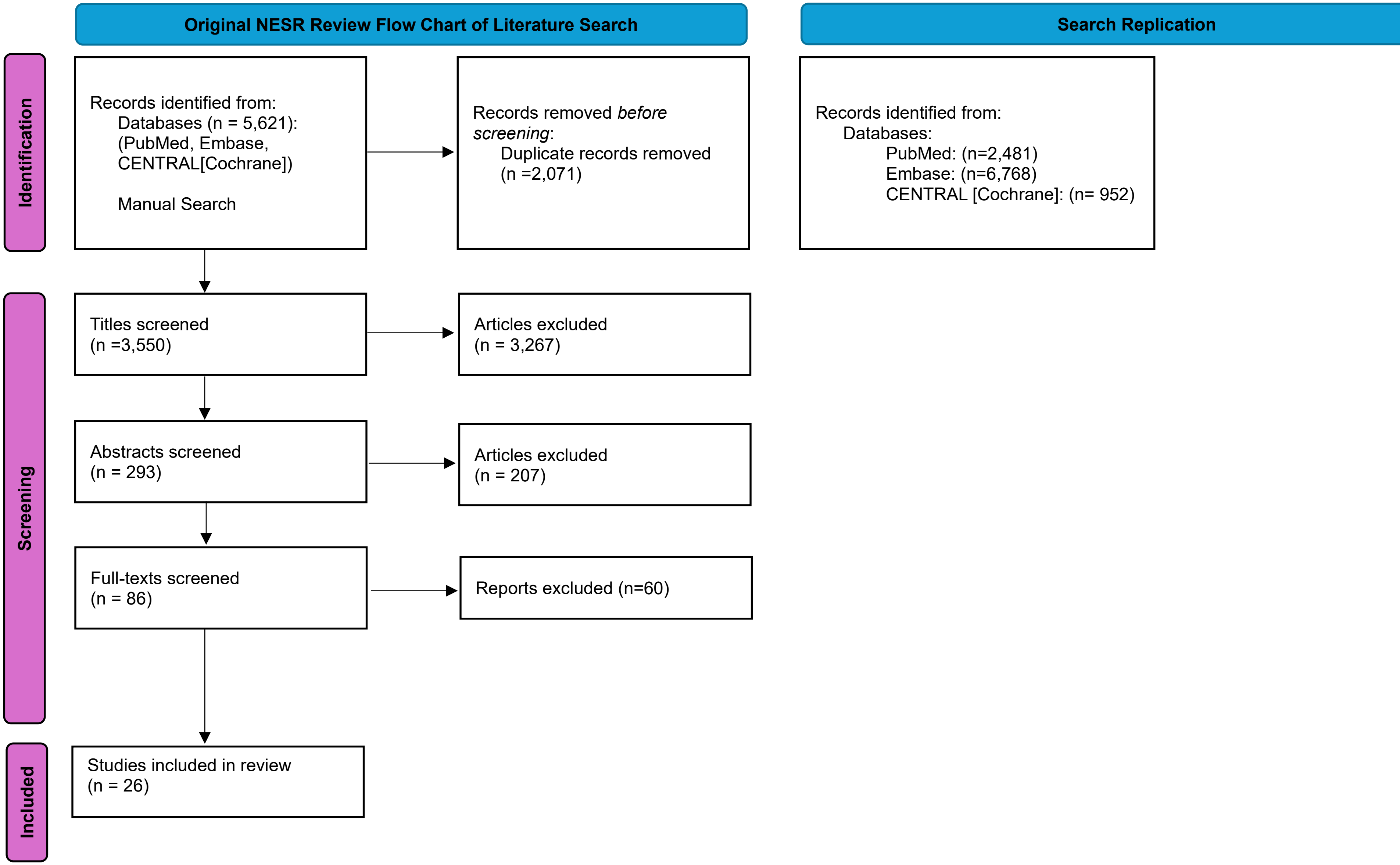

PRISMA Flowchart from: Page MJ, McKenzie JE, Bossuyt PM, Boutron I, Hoffmann TC, Mulrow CD, et al. The PRISMA 2020 statement: an updated guideline for reporting systematic reviews. BMJ 2021;372:n71. doi: 10.1136/bmj.n71.
NESR results from: Boushey, C.; Ard, J.; Bazzano, L.; Heymsfield, S.; Mayer-Davis, E.; Sabaté, J.; Snetselaar, L.; Van Horn, L.; Schneeman, B.; English, L.; et al. Dietary Patterns and Neurocognitive Health: A Systematic Review . **2020**, doi:10.52570/NESR.DGAC2020.SR0106, Figure 2: Flow chart of literature search and screening results

Supplementary File 7

PRESS Guideline — Search Submission & Peer Review Assessment

1. Systematic Review Title

Dietary Patterns and Neurocognitive Health: A Systematic Review

2. This search strategy is (highlight the appropriate response):

A. My PRIMARY (core) database strategy — First time submitting a strategy for search question and database
B. My PRIMARY (core) strategy — Follow-up review NOT the first time submitting a strategy for search question and database. If this is a response to peer review, itemize the changes made to the review suggestions
C. SECONDARY search strategy— First time submitting a strategy for search question and database
D. SECONDARY search strategy – NOT the first time submitting a strategy for search question and database. If this is a response to peer review, itemize the changes made to the review suggestions.

3. Database (e.g., Medline, CINAHL)

PubMed

4. Interface (e.g.,Ovid, EBSCO)

pubmed.ncbi.nlm.nih.gov

5. Research Question. Describe the purpose of the search

What is the relationship between dietary patterns consumed and neurocognitive health?

6. PICO Format (Outline the PICOs for your question – ie. Patient, Intervention, Comparison, Outcome and Study Design – as applicable)

P	What is the population of interest?<br>• For the intervention/exposure, children through older adults, age 2 years and older<br>• For the outcome, adults and older adults, age 19 years and older
I	Dietary consumption

C	
O	Neurocognitive health

Inclusion Criteria (List criteria such as age groups, study designs, etc, to be included) [optional]

Exclusion Criteria (List criteria such as study designs, date limits, etc., to be excluded) [optional]

7. Was a search filter applied?

No

If YES, which one(s) (e.g., Cochrane RCT filter, PubMed Clinical Queries filter)? Provide the source if this is a published filter. [mandatory if YES to previous question — textbox]

N/A

Other notes or comments you feel would be useful for the peer reviewer? [optional]

Please copy and paste your search strategy here, exactly as run, including the number of hits per line. [mandatory]

PubMed

#1 - dietary pattern* OR diet pattern* OR eating pattern* OR food pattern* OR diet quality* OR eating habit* OR dietary habit* OR diet habit* OR food habit* OR beverage habit* OR "Feeding Behavior"[Mesh:NoExp] OR feeding behavior*[tiab] OR dietary profile* OR food profile* OR diet profile* OR eating profile* OR dietary guideline* OR dietary recommendation* OR dietary intake* OR eating style* OR "Diet, Mediterranean"[Mesh] OR Mediterranean Diet*[tiab] OR "Dietary Approaches To Stop Hypertension"[Mesh] OR Dietary Approaches To Stop Hypertension Diet* OR DASH diet* OR "Diet, Gluten-Free"[Mesh] OR Gluten Free diet* OR prudent diet* OR "Diet, Paleolithic"[Mesh] OR Paleolithic Diet* OR "Diet, Vegetarian"[Mesh] OR vegetarian diet*[tiab] OR vegan diet* OR "Diet, Healthy"[Mesh] OR healthy diet* OR plant based diet* OR "Diet, Western"[Mesh] OR western diet* OR "Diet, Carbohydrate-Restricted"[Mesh] OR low-carbohydrate diet* OR high carbohydrate diet* OR Ketogenic Diet* OR Nordic

Commented [VL1]: Nearly all the MeSH selected for this search line all fall under the major heading of "diet". Most of the subheadings were selected that related to a food type, except "Diet, Carbohydrate Loading", which may have accidently been missed. It might be worth just searching the MeSH for diet and leaving it exploded, so that the concept is searched comprehensively.

Commented [VL2]: These multi-word terms would need to be in quotation marks or else a term like dietary pattern* will be term mapped to (("diet"[MeSH Terms] OR "diet"[All Fields] OR "dietary"[All Fields] OR "dietaries"[All Fields]) AND "pattern*"[All Fields]).
These terms should also be restricted to [tiab] like some of the latter terms or it will be searched in all fields.
Based on these suggestions, this should be searched as: "dietary pattern*"[tiab] OR "diet pattern*"[tiab] OR "eating pattern*"[tiab] OR "food pattern*"[tiab] OR "diet quality*"[tiab] OR "eating habit*"[tiab] OR "dietary habit*"[tiab] OR "diet habit*"[tiab] OR "food habit*"[tiab] OR "beverage habit*"[tiab]

Commented [VL3]: Needs quotation marks around terms.

Commented [VL4]: Needs to be limited to [tiab]

Commented [VL5]: Needs quotation marks around term.

Commented [VL6]: Needs quotation marks around terms and [tiab].

Commented [VL7]: Needs quotation marks around terms and [tiab].

Commented [VL8]: Needs quotation marks around term and [tiab].

Commented [VL9]: Needs quotation marks around terms.

Commented [VL10]: Limit to [tiab].

Commented [VL11]: Needs quotation marks around terms and [tiab].

Commented [VL12]: Needs quotation marks around term and [tiab].

Diet* OR "Diet, Fat-Restricted"[Mesh] OR "Diet, High-Fat"[Mesh] OR "Diet, High-Protein"[Mesh] OR high protein diet*[tiab] OR protein intake* OR high-fat diet* OR low fat diet* OR "Diet, Protein-Restricted"[Mesh] OR low protein diet* OR "Diet, Sodium-Restricted"[Mesh] OR low-sodium diet* OR low salt diet* OR ((“Guideline Adherence"[Mesh] OR guideline adherence*) AND (diet[tiab] OR dietary[tiab] OR food[tiab] OR beverage*[tiab] OR nutrition*[tiab])) OR diet score* OR diet quality score* OR diet quality index* OR kidmed OR diet index* OR dietary index* OR food score* OR MedDietScore OR healthy eating index[tiab] OR ((pattern[tiab] OR patterns[tiab] OR consumption[tiab] OR habit*[tiab]) AND (“Diet"[Mesh:NoExp] OR diet[tiab] OR diets[tiab] OR dietary[tiab] OR "Food"[Mesh] OR food[tiab] OR foods[tiab] OR "Beverages"[Mesh] OR beverage[tiab] OR beverages[tiab]))

#2 - "Cognition Disorders"[Mesh] OR "Cognition"[Mesh] OR cognition[tiab] OR metacognition[tiab] OR neurocognitive[tiab] OR "Dementia"[Mesh] OR dementia[tiab] OR Alzheimer*[tiab] OR senility[tiab] OR senile[tiab] OR presenile[tiab] OR (cognit*[tiab] AND (function*[tiab] OR dysfunction*[tiab] OR declin*[tiab] OR deteriorat* OR degenerat*[tiab] OR disorder*[tiab] OR dysfunction*[tiab] OR reduct*[tiab] OR impair*[tiab] OR deficit*[tiab] OR deficien* OR progress*[tiab] OR perform*[tiab] OR abilit*[tiab]))

#3 - (#1 AND #2)

#4 - (#1 AND #2) NOT ("Animals"[Mesh] NOT ("Animals"[Mesh] AND "Humans"[Mesh])) NOT (editorial[ptyp] OR comment[ptyp] OR news[ptyp] OR letter[ptyp] OR review[ptyp] OR systematic review[ptyp] OR systematic review[ti] OR meta-analysis[ptyp] OR meta-analysis[ti] OR meta-analyses[ti] OR retracted publication[ptyp] OR retraction of publication[ptyp] OR retraction of publication[tiab] OR retraction notice[ti]) Filters: Publication date from 2014/01/01 to 2020/02/04; English

Commented [VL13]: Needs quotation marks around terms and [tiab].

Commented [VL14]: Needs quotation marks around terms.

Commented [VL15]: Needs to be limited to [tiab].

Commented [VL16]: Needs quotation marks around term and [tiab].

Commented [VL17]: Needs quotation marks around terms and [tiab].

Commented [VL18]: Needs quotation marks around term and [tiab].

Commented [VL19]: I would search this as diet*[tiab] instead so we can capture diet, diets, dietary, dieter, dieters, etc.

Commented [VL20]: Would search as food*[tiab] to capture foods as well, or alternatively search food[tiab] OR foods[tiab]

Commented [VL21]: Needs quotation marks around terms and [tiab].

Commented [VL22]: Needs to be limited to [tiab].

Commented [VL23]: Needs quotation marks around terms and [tiab].

Commented [VL24]: Needs to be limited to [tiab].

Commented [VL25]: Needs quotation marks around term.

Commented [VL26]: This section has some redundancy to other search terms in line 1. For example dietary guideline, dietary pattern, dietary habit, etc. are searched earlier. I prefer this latter search because it will be more comprehensive, so I think some of the earlier terms should be removed. I do believe that this section should be further developed though.

Commented [VL27]: All the MeSH in this search line are exploded to include a lot of subheadings that aren't named in this keyword search strategy. I would reevaluate wheth ...

Commented [VL28]: I would maybe truncate this earlie ...

Commented [VL29]: I would truncate these to ...

Commented [VL30]: In the MeSH listing for Dementia, ...

Commented [VL31]: Maybe include the word mental* ...

Commented [VL32]: Maybe add the keyword lost, loss ...

Commented [VL33]: This was searched already, so it's ...

Commented [VL34]: I would truncate this earlier, so it' ...

Commented [VL35]: Needs to be limited to [tiab].

Commented [VL36]: The exclusion of specific publicati ...

PEER REVIEW ASSESSMENT: THIS SECTION TO BE FILLED IN BY THE REVIEWER

Reviewer: Valentina Ly and Catherine Gracey	Email: vly@uottawa.ca and cgracey@dal.ca	Date Completed:2023-08-17

1. Translation

A - No Revision

2. Boolean and Proximity Operators

A - No Revision

3. Subject Headings

B - Revision suggestion

If “B’ or “C”, please provide and explanation or example
Subject headings should be reevaluated as to whether to explode subheadings or not. Please see comments for further revision suggestions for subject headings.

4. Text Word Searching

C - Revision Required

If “B’ or “C”, please provide and explanation or example
Please see notes for suggestions regarding additional keywords to include and suggestions for better truncation options.

5. Spelling, Syntax and Line Numbers

C - Revision Required

If “B’ or “C”, please provide and explanation or example
Field tags for title and abstract [tiab] were inconsistently applied to text words and many text words needed to be searched in quotation marks to prevent PubMed’s automatic term mapping.

6. Limits and Filters

B - Revision suggestion

If “B’ or “C”, please provide and explanation or example

The human filter in line 4 is appropriate, but the exclusion of specific publication types (i.e., editorials, comments, news, letters, systematic and narrative reviews, retracted publication and retraction notices) seems unnecessary to include since it could remove potentially relevant articles. I would recommend having the team screen out those publication types to ensure the review is comprehensive.

7. Overall Evaluation

C - Revision Required

Additional Comments:

There are several issues and inconsistencies regarding syntax throughout the search strategy that greatly affect it, especially the inconsistent title and abstract field tags. Some of the search terms are redundant and can be removed to simplify the search strategy. Be aware of hyphens between words, for example “high-fat diet” when translating to other databases. Hyphens don’t affect the PubMed search, but the presence of hyphens affects the Cochrane Wiley search.
